# DNA replication dynamics of vole genome and its epigenetic regulation

**DOI:** 10.1186/s13072-019-0262-0

**Published:** 2019-03-14

**Authors:** Kathrin S. Heinz, Alexander Rapp, Corella S. Casas-Delucchi, Anne Lehmkuhl, Ismael Romero-Fernández, Antonio Sánchez, Oliver H. Krämer, J. Alberto Marchal, M. Cristina Cardoso

**Affiliations:** 1 Cell Biology and Epigenetics, Department of Biology, Technische Universität Darmstadt, Schnittspahnstrasse 10, 64287 Darmstadt, Germany; 2 Department of Experimental Biology, University of Jaén, Jaén, Spain; 3 Institute of Toxicology, Universitätsmedizin der Johannes Gutenberg-Universität Mainz, Mainz, Germany; 4 Present Address: Chromosome Replication Laboratory, The Francis Crick Institute, London, NW1 1AT UK

**Keywords:** Heterochromatin, *Microtus cabrerae*, Giant sex chromosomes, Epigenetic composition, Histone acetylation, HDAC inhibition, Site-directed targeting, DNA replication dynamics

## Abstract

**Background:**

The genome of some vole rodents exhibit large blocks of heterochromatin coupled to their sex chromosomes. The DNA composition and transcriptional activity of these heterochromatin blocks have been studied, but little is known about their DNA replication dynamics and epigenetic composition.

**Results:**

Here, we show prominent epigenetic marks of the heterochromatic blocks in the giant sex chromosomes of female *Microtus cabrerae* cells. While the X chromosomes are hypoacetylated and cytosine hypomethylated, they are either enriched for macroH2A and H3K27me3 typical for facultative heterochromatin or for H3K9me3 and HP1 beta typical for constitutive heterochromatin. Using pulse-chase replication labeling and time-lapse microscopy, we found that the heterochromatic block enriched for macroH2A/H3K27me3 of the X chromosome is replicated during mid-S-phase, prior to the heterochromatic block enriched for H3K9me3/HP1 beta, which is replicated during late S-phase. To test whether histone acetylation level regulates its replication dynamics, we induced either global hyperacetylation by pharmacological inhibition or by targeting a histone acetyltransferase to the heterochromatic region of the X chromosomes. Our data reveal that histone acetylation level affects DNA replication dynamics of the sex chromosomes’ heterochromatin and leads to a global reduction in replication fork rate genome wide.

**Conclusions:**

In conclusion, we mapped major epigenetic modifications controlling the structure of the sex chromosome-associated heterochromatin and demonstrated the occurrence of differences in the molecular mechanisms controlling the replication timing of the heterochromatic blocks at the sex chromosomes in female *Microtus cabrerae* cells. Furthermore, we highlighted a conserved role of histone acetylation level on replication dynamics across mammalian species.

**Electronic supplementary material:**

The online version of this article (10.1186/s13072-019-0262-0) contains supplementary material, which is available to authorized users.

## Background

In higher eukaryotes, the nuclear genome is compartmentalized into distinct chromatin territories to facilitate the regulation of complex processes such as DNA repair, transcription and replication. The DNA replication process is highly regulated both spatially and temporally, resulting in the changing pattern of replication structures throughout S-phase. The temporal order of DNA replication reflects this higher-order organization of the nuclear genome [1–3]. Eu- and heterochromatin, as major higher-order chromatin structures, are defined by a complex interplay between their condensation state, chromatin modifications, associated proteins, as well as their transcriptional activity, all referred to as epigenetic marks [4–6]. These epigenetic properties of chromatin regions are potential determinants of their DNA replication timing [7–10]. In mammals, constitutive heterochromatin is mostly arranged at pericentromeric regions of the chromosomes, whereas vole rodents (subfamily *Arvicolinae*) are a remarkable exception. In some vole species, a bulk of constitutive heterochromatin is coupled to both sex chromosomes. These enlarged X and Y chromosomes are referred to as “giant” sex chromosomes [11, 12] and represent an interesting biological model to study the basis of heterochromatin organization and dynamics in a different genomic context.

Euchromatin includes less-condensed transcriptional active regions, determined by a depletion of methylated DNA, an enrichment in specifically methylated histones such as H3K4, H3K36, H3K79 and a high level of histone acetylation [13]. Euchromatin is replicated in early S-phase, when the replication machinery is present as a multitude of small replication foci that are well distributed throughout the nuclear interior. This is followed by the DNA replication of facultative heterochromatin, which corresponds to developmentally silenced regions that are enriched in H3K27 trimethylation (H3K27me3) with the inactive X chromosome in mammals as one of the most prominent examples [7, 14, 15]. This chromatin is often also enriched for macroH2A variant histones, which are not only abundant on the inactive X chromosome but also on developmentally regulated regions bound by polycomb repressive complexes and, thus, marked by H3K27me3 [16, 17]. This type of heterochromatin is replicated during the mid-S-phase, when the replication foci become larger and are located around the nucle(ol)ar periphery. The second type of heterochromatin, termed constitutive heterochromatin, is replicated in the late S-phase. This type of heterochromatin is enriched for a set of histone modifications such as H3K9 and H4K20 trimethylation and is histone hypoacetylated [18]. H3K9 trimethylation is recognized and bound by the non-histone protein chromodomain-containing heterochromatin protein 1 (HP1) [19], which is consequently accumulated at heterochromatic regions. Several lines of evidence support the idea of an interaction of epigenetic properties and DNA replication timing of a given genomic region. For instance, manipulations in yeast showed that the deletion of the histone deacetylase (HDAC) Rpd3 led to an increased acetylation level at many replication origins and subsequently to an earlier onset of DNA replication [20]. Studies in human cells congruently illustrated that a treatment with the HDAC inhibitor trichostatin A (TSA) resulted in an early initiation of DNA replication of imprinted genes [21, 22]. Furthermore, manipulations of epigenetic marks of the well-defined and prominent heterochromatic landscape of pericentromeric regions in mouse cells demonstrated that histone hypoacetylation was required to maintain the characteristic late DNA replication timing of constitutive heterochromatin [9]. In addition, histone hypoacetylation was shown to have a key role in controlling the DNA replication dynamics of the inactive X chromosome in mouse cells [7].

In this study, we assess the DNA replication dynamics in female cells from the vole species *Microtus cabrerae*, a species presenting giant sex chromosomes with enlarged heterochromatin blocks and, thus, test the validity and reproducibility of the epigenetic control of replication dynamics across mammalian species. We first determine the epigenetic constitution of the sex chromosome-associated heterochromatic blocks and second dissect the timing and regulation of DNA replication of the different chromatin states. Our study revealed that histone hypoacetylation and strikingly also DNA demethylation are major epigenetic determinants of this sex heterochromatin. We distinguished between X chromosome-associated heterochromatin block enriched for H3K27me3 and macroH2A, and X chromosome-associated heterochromatin block exhibiting H3K9me3 and HP1 accumulation. We found that the former replicated before the latter, and each heterochromatin block replicated rather synchronously. To test whether histone acetylation levels might be involved in setting up the synchronous DNA replication process, we manipulated histone acetylation globally by HDAC inhibition or by site-directed HAT targeting. We showed that an increase in histone acetylation levels affects DNA replication dynamics and leads to a prolongation of total and early S-phase, as well as of X chromosome-associated heterochromatin block replication. Finally, we found a global decrease in replication fork speed in hyperacetylated cells going hand in hand with a prolongation of S-phase.

## Results and discussion

### Subnuclear distribution of euchromatin and heterochromatin marks in female *Microtus cabrerae* fibroblasts

**Fig. 1:**
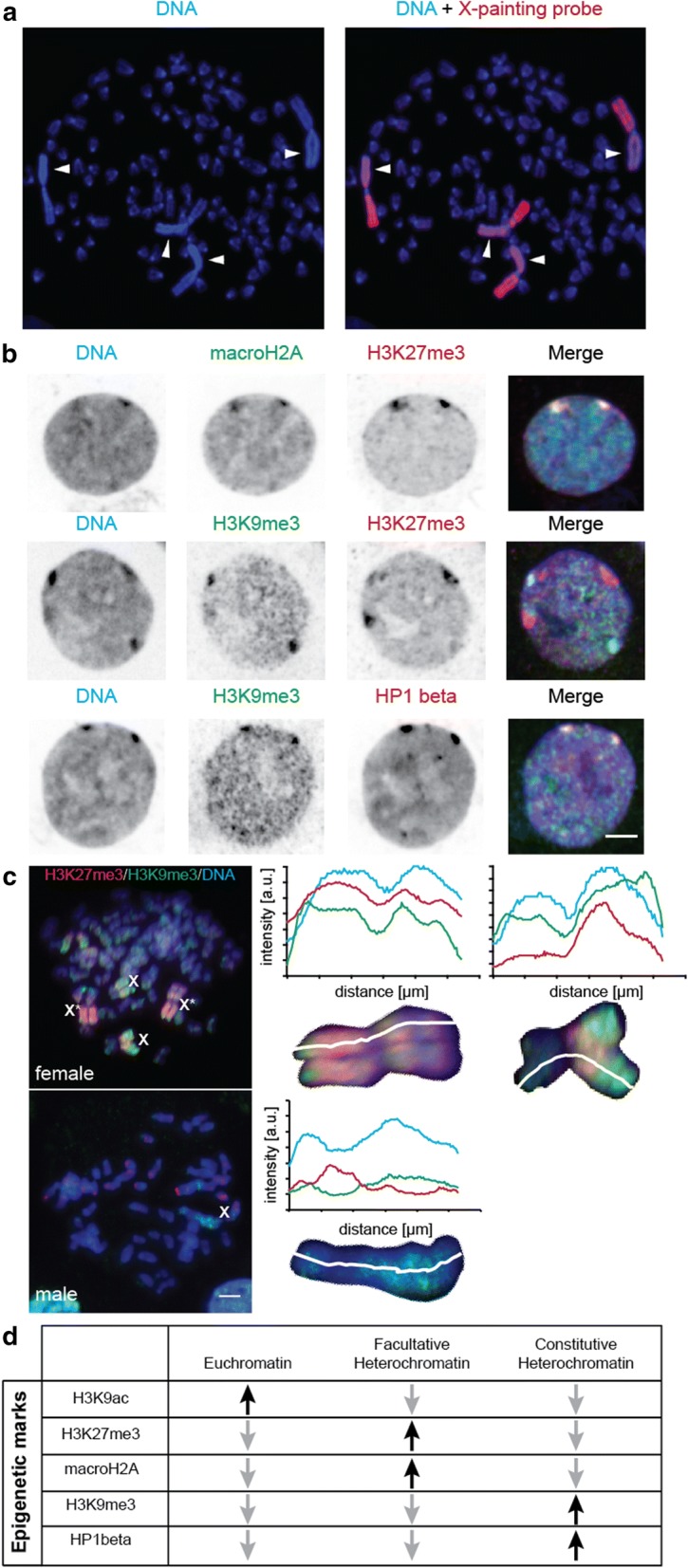
Subnuclear distribution of facultative and constitutive heterochromatin marks in female *Microtus cabrerae* fibroblasts. **a** Metaphase chromosomes from female *Microtus cabrerae* cell line were analyzed with a painting probe from the X chromosome. Arrow points to the heterochromatic block, which occupies the whole short arm of the X chromosome. **b** Prominent chromatin marks were analyzed by transient transfections and immunostaining. MacroH2A1 and H3K27me3 signals were simultaneously visualized by transient transfection with GFP-macroH2A1 and immunostaining using anti-H3K27me3 antibody (upper row). H3K9me3 and H3K27me3 marks, typical for constitutive and facultative heterochromatin, were simultaneously analyzed by double immunostaining (middle row). HP1 beta and H3K9me3 signals were simultaneously visualized by transient transfection with DsRed-HP1 beta and immunostaining against H3K9me3 (lower row). DNA was counterstained with DAPI. Protein and merge signals of all channels are shown. Single optical sections acquired on a spinning disk confocal microscope are shown. Scale bar 5 µm. **c** Immunofluorescence detection of histone H3 posttranslational modifications on metaphase chromosomes. Upper row female *Microtus cabrerae* cells (tetraploid) show two pairs of either H3K27me3 or H3K9me3 decorated giant sex chromosomes. The line plots along the enlarged metaphase sex chromosomes depict the fluorescence intensity distribution of the H3K27me3-enriched sex chromosome (X*) and the H3K9me3-enriched sex chromosome (X). In contrast, in the male *Microtus cabrerae* cell line (nearly diploid) only one giant X chromosome is found that is enriched in H3K9me3, especially on the long arm. Scale bar 5 µm. **d** Overview of subnuclear distribution of facultative and constitutive heterochromatin marks in female *Microtus cabrerae* cell line. Euchromatin is enriched for euchromatic marks such as H3K9ac and H4K8ac, but depleted for heterochromatin marks such as H3K27me3 and H3K9me3. Facultative heterochromatin is enriched for H3K27 trimethylation and macroH2A1. The constitutive heterochromatin is characterized by marks such as H3K9me3 and HP1 beta accumulation

In a previous study, we showed that in cell lines derived from male voles of two *Microtus* species (*M. agrestis* and *M. cabrerae*), the heterochromatic blocks from the sex chromosomes are often visible during interphase as bright dense regions of DAPI-stained chromatin [23]. Here, we have made use of this feature to investigate the epigenetic composition of the heterochromatic block coupled to the X chromosomes of female *Microtus cabrerae* cells. Since four DAPI bright regions were visible per cell, we controlled the karyotype of the cell line by metaphase spreading followed by chromosome painting using a specific probe for the X chromosome (Fig. 1a) [24]. This result indicated, as suspected, the tetraploid condition of the cells, with four giant X chromosomes being included in one single nucleus. Next, we made use of double immunostaining using antibodies specific for facultative (H3K27me3) and constitutive (H3K9me3) heterochromatin. As depicted in Fig. 1b, each mark produced an intense signal onto one heterochromatic block. However, the prominent signals of each mark did not colocalize. As we employed optical sectioning microscopy, sometimes not all four heterochromatic blocks are seen in the Z plane shown. We, then, analyzed in metaphase chromosomes the distribution of both H3K9me3 and H3K27me3 by performing double immunostaining in metaphase chromosomes and compared the signals in male versus female *Microtus cabrerae* cells. To obtain antibody staining, we needed to avoid the rather harsh conditions used for metaphase spreading, and consequently, the chromosome signals and morphology are less well resolved. Notwithstanding this caveat, whereas in male cells there was one larger chromosome (X) partly labeled by H3K9me3, in female cells we found, in addition, X chromosomes (X*) marked with H3K27me3 marks along their length (Fig. 1c). Based on this metaphase analysis, it is not possible to more precisely determine how much of each chromosome was labeled with each of the two marks. Taking into consideration the analysis in interphase cells, the two blocks marked with facultative and constitutive marks did not show appreciable overlap (Fig. 1c). To further characterize and discriminate the two different types of heterochromatic blocks in *Microtus cabrerae* cells, we extended our analysis for chromatin marks typical for facultative or constitutive heterochromatin. First, we transiently transfected GFP-tagged histone variant macroH2A1 in cells, where H3K27me3 was simultaneously detected via immunodetection. Both signals clearly colocalized in the same heterochromatic blocks (two of four heterochromatic blocks). Similarly, in cells transiently transfected with GFP-tagged HP1 beta and simultaneously stained for H3K9me3, enrichment for both signals in the same heterochromatic blocks was observed. Furthermore, we also investigated several euchromatic marks such as H3K4 dimethylation (H3K4me2), H4K8 acetylation (H4K8ac), H3K9 acetylation (H3K9ac), as well as the level of DNA methylation (Additional file 1A). As shown before for male vole cells [23], the heterochromatic blocks from the X chromosomes of female *Microtus cabrerae* cells were depleted of euchromatic marks and their DNA was hypomethylated. In a previous study in male cells from voles, these repeats were shown to be transcribed [23]. Hence, we performed transcription run-on analysis and measured the relative amount of nucleotide incorporation within the differently marked heterochromatic blocks. We found a small, but significant, lower transcription within the H3K27me3-enriched block relative to the H3K9me3-enriched block (Additional file 1B).

Altogether, our results indicate that half of the heterochromatic blocks were enriched in the H3K27me3/macroH2A signal (“facultative” heterochromatic block), while the other half showed accumulation of H3K9me3/HP1 beta signal (“constitutive” heterochromatic block) (Fig. 1d). This organization is different to that observed in another *Microtus* species, *Microtus rossiameridionalis* [25]. In this case, the epigenetic marks at the heterochromatic blocks of both X chromosomes were similar. Interestingly, early studies suggest that the timing of replication of the heterochromatic blocks from *Microtus cabrerae* could be different [26]. Therefore, we next studied the DNA replication dynamics of the heterochromatic blocks in female *Microtus cabrerae* cells.

### Heterochromatic blocks from the differently epigenetically marked X chromosomes replicate at distinct times

**Fig. 2:**
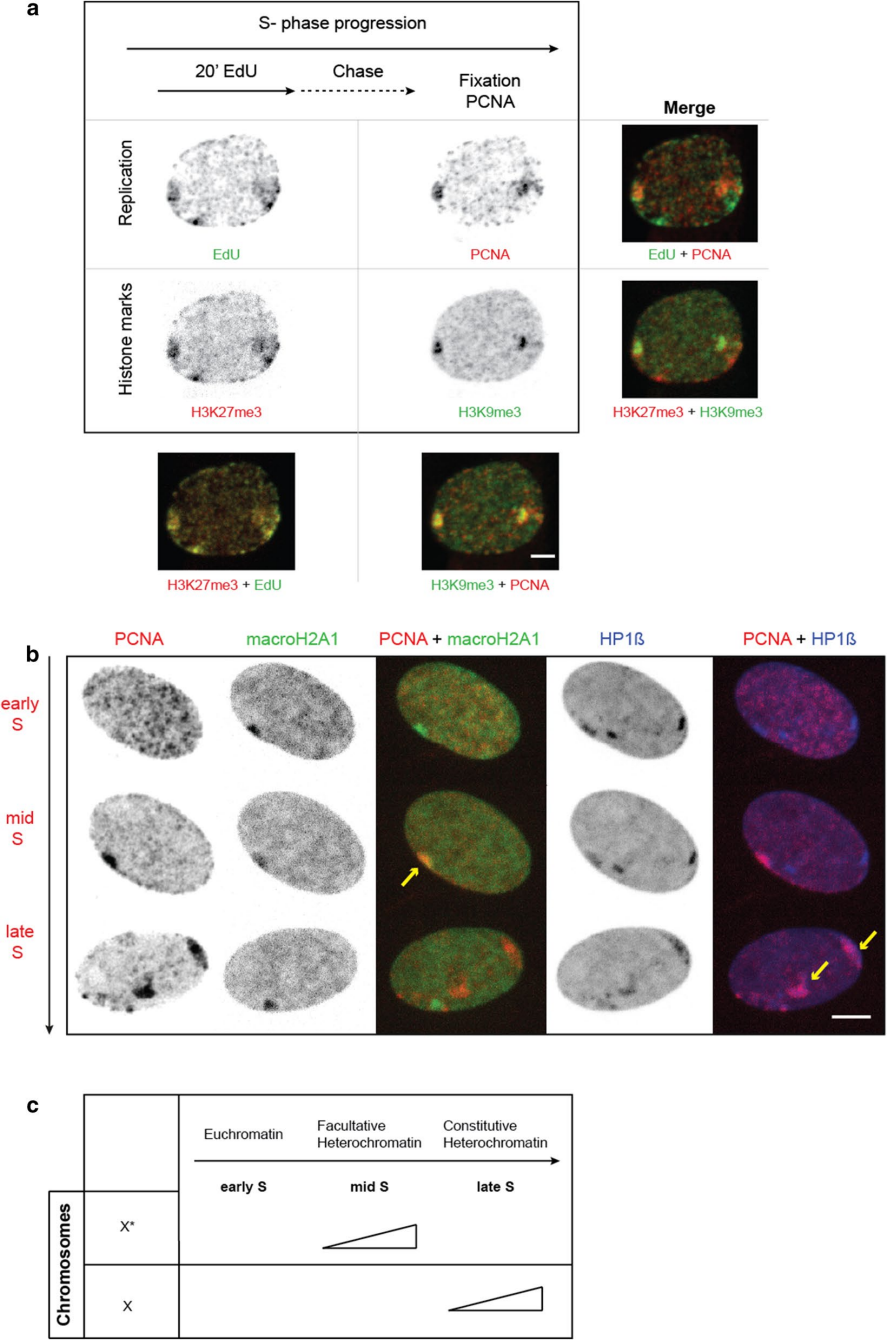
DNA replication dynamics and epigenetic constitution of facultative and constitutive heterochromatin in female *Microtus cabrerae* fibroblasts. **a** Schematic representation of the experimental strategy: Asynchronously growing cultures of female *Microtus cabrerae* cells were transfected with CFP-PCNA plasmid and pulse labeled with 10 μM EdU for 20 min. This nucleotide pulse was followed by a 1-h chase before fixation with 4% paraformaldehyde and methanol. EdU was detected with the ClickIT system with Alexa488 followed by immunostaining against H3K27me3 (facultative heterochromatin), H3K9me3 (constitutive heterochromatin) and PCNA to visualize ongoing replication at the time of fixation. This strategy allowed us to determine two time points of S-phase in every cell, which are 1 h apart determined by the chase period. The combination with the two histone mark antibody staining permits then the identification of the replication order of the chromatin marked with one versus the other histone modification. The data indicated that the H3K27me3-enriched heterochromatic block is replicated prior to the H3K9me3-enriched heterochromatic block. Maximum intensity projections of confocal images are shown. Scale bar 5 μm. **b** Live images from female *Microtus cabrerae* cells triple transfected with CFP-PCNA, GFP-macroH2A1 and DsRedHP1 beta. Maximum intensity projection of z-stacks acquired on a spinning disk confocal microscope at 20-min time intervals. Yellow arrows denote ongoing replication of the heterochromatic blocks of the chromosomes. Exemplary images depict three distinct PCNA patterns, which can be assigned to three different types of chromatin. In early S-phase, a multitude of small replication foci was distributed throughout the whole nucleus, excluding the nucleolus, when euchromatin was replicated. In mid-S-phase, the replication foci became more organized and a perinuclear pattern arises, with foci at the nucle(ol)ar periphery. A first big blob was identified colocalizing with the heterochromatic block of the X chromosome, which is enriched for macroH2A1, whereas in late S-phase the replication foci were consolidated together in large blobs of replication foci colocalizing with the heterochromatic block of the X chromosome, which is enriched in HP1 beta. Both heterochromatic blocks were replicated later than the euchromatin. The orientation of the cell at the end of the time lapse is different due to cell movement over the hours of imaging. Scale bar 5 μm. For full time-lapse, see Additional file 2 Movie 1. **c** Summary of the replication timing of the H3K9me3- (X) and the H3K27me3 (X*)-enriched heterochromatic blocks of the X chromosomes

To dissect the DNA replication dynamics of the heterochromatic blocks in female *Microtus cabrerae* cells, we transiently transfected cells with a construct encoding for CFP-PCNA to label active replication sites. Simultaneously, we performed in situ replication labeling in combination with immunodetection of the previously characterized histone marks H3K27me3 and H3K9me3 (Fig. 2a). Modified nucleotides (EdU) were added to proliferating populations of cells prior to fixation, with a labeling pulse of 20 min followed by a chase of 1 h. Ongoing DNA replication at the time of fixation, i.e., 1 h after the nucleotide pulse ended, was identified through CFP-PCNA labeling of replication sites avoiding the need for a second and distinct nucleotide pulse. Such pulse-chase experiments allow to label in every S-phase cell two distinct and consecutive times of S-phase, which, when combined with the histone mark detection, yields the order of replication of the differently marked chromatin. Hence, with this strategy we could distinguish between the DNA replication of the heterochromatic blocks coupled to the X enriched in H3K27me3, or the X enriched in H3K9me3. After a chase of 1 h (Fig. 2a), we detected an overlap of EdU and H3K27me3 in the first pulse but, at the time point of fixation, there was only colocalization of H3K9me3 and PCNA left. This indicated that both heterochromatin types were replicated at distinct times during S-phase with the heterochromatin enriched in H3K27me3 mark being replicated first and later on the heterochromatin enriched for H3K9me3 marks.

### Spatio-temporal progression of DNA replication in female *Microtus cabrerae* cells

For a more detailed analysis of the previous results, we next studied the spatio-temporal progression of the DNA replication process in female *Microtus cabrerae* cells. To this end, we performed time-lapse microscopy of these cells, triple transfected with constructs encoding for CFP-PCNA, GFP-macroH2A1 and DsRed-HP1 beta (Fig. 2b, Additional file 3). With the help of PCNA, we identified DNA replication patterns (early, mid and late), while macroH2A1 was used to detect the facultative heterochromatic block and with HP1 beta we spotted the constitutive heterochromatic block. In early S-phase, a multitude of small replication foci was distributed throughout the whole nucleus, excluding the nucleoli and the X chromosomes. This pattern was clearly comparable to the early S-phase pattern in other mammals, when euchromatin is replicated. With S-phase progression (4 h), another pattern arose, which showed more organized replication foci localized perinuclearly. The first big blob appeared in this phase, colocalizing with macroH2A1, indicating the DNA replication of the facultative heterochromatic block. After additional 3 h, there was no longer a colocalization of PCNA with macroH2A1, indicating the progression from mid-to-late S-phase. Now, there was a strong colocalization of HP1 beta and PCNA within two big perinuclear blobs as a third distinct pattern of S-phase. As this structure colocalized with HP1 beta, we identified this structure as the constitutive heterochromatic block. From these three distinct patterns, we concluded that the DNA replication timing follows the chromatin state (Fig. 2c). First, euchromatin followed by facultative heterochromatin and lastly constitutive heterochromatin were duplicated. These results clearly confirm that the timing of replication of the epigenetically differently marked heterochromatic blocks is dissimilar in female *Microtus cabrerae* cells, in line with early evidence [26] and establish their order of replication during S-phase.

### Treatment with HDAC inhibitor induces global histone hyperacetylation as well as at the heterochromatic blocks of the X chromosomes

Next, we tested histone acetylation as a potential regulator of DNA replication dynamics in the vole rodent, because earlier studies have shown that histone acetylation level has an impact on DNA replication dynamics. Histone acetylation was shown to control the DNA replication dynamics of the inactive X chromosome [7] and to be required to maintain the late DNA replication timing of constitutive heterochromatin in murine C2C12 cells [9]. As we identified that both types of heterochromatic blocks were hypoacetylated, we tested whether this mark regulated DNA replication timing.

First, we investigated whether treatment of cells with a histone deacetylase inhibitor (HDACi) induces global hyperacetylation (Additional file 4). Cells were either treated with HDACi (LBH-589, panobinostat [27]) for 24 h or with DMSO only and, afterward, subjected to either immunofluorescence or to live-cell imaging analysis. Other HDAC inhibitors (TSA, MS-275) proved less efficient and more toxic (Additional file 6). We developed a self-written analysis protocol to measure mean acetylation level in the whole nucleus (Additional file 5). Our results showed a significant increase in histone acetylation level in cells treated with HDACi, demonstrating an efficient induction of global hyperacetylation (Additional file 4B).

**Fig. 3:**
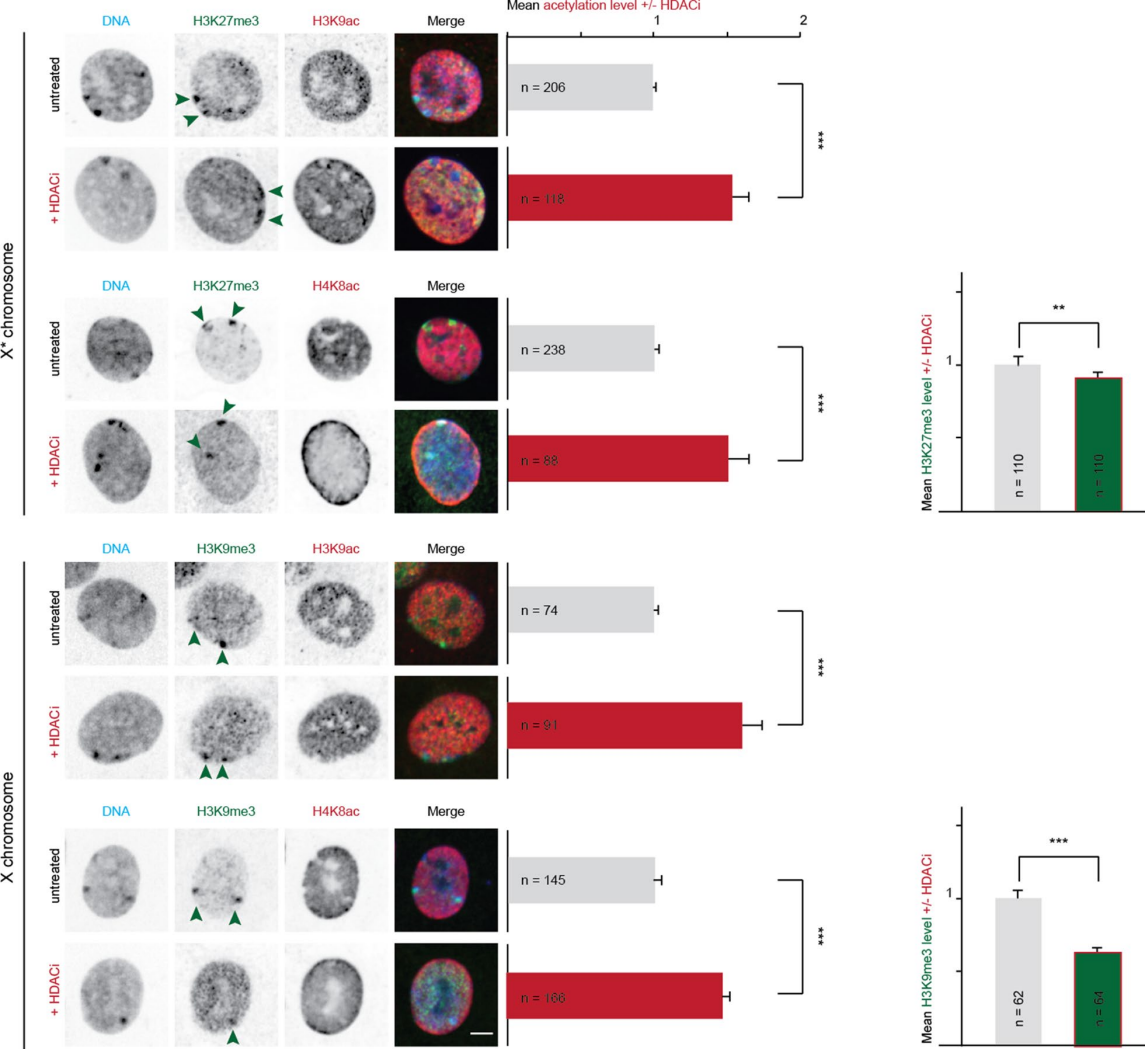
Treatment with HDAC inhibitor leads to hyperacetylation of heterochromatic blocks at sex chromosomes and decrease in methylation marks. Distribution of euchromatic marks H3K9ac and H4K8ac was analyzed by immunofluorescence staining. Female *Microtus cabrerae* cells were treated with or without HDACi LBH-589 (50 nM) for 24 h prior fixation. Acetylation levels were measured with a user-independent analysis: DNA (DAPI, blue), X*/X (H3K27me3/H3K9me3, green), acetylation (H3K9ac or H4K8ac, red). Arrows depict heterochromatic blocks at the H3K27me3 and H3K9me3 decorated X chromosomes. Mean acetylation signals for untreated cells (gray bar) and HDACi-treated cells (red bar) are shown. Sample sizes are indicated in the bar. Mean methylation levels are plotted for untreated cells (gray bar) and for HDACi-treated cells (green bar, red-framed). Scale bar 5 µm. Error bars demonstrate 95 Cl. ***P < 0.001

We next investigated whether the treatment with the HDAC inhibitor was sufficient to affect the heterochromatic blocks of the sex chromosomes. We analyzed the histone acetylation level directly at the heterochromatic blocks (Fig. 3) by using the analysis protocol described in Additional file 5. We estimated the acetylation levels at the heterochromatin enriched in either H3K27me3 or H3K9me3 in cells treated with the HDACi. We measured two different acetylation marks, H3K9ac and H4K8ac, which were depleted from the heterochromatic blocks (Additional file 1). We observed for H3K27me3-enriched heterochromatin that both acetylation marks were significantly increased in cells treated with HDACi in contrast to untreated cells (Fig. 3). Similar results were also obtained for H3K9me3-enriched heterochromatin. Interestingly, H4K8ac was more pronounced at the H3K27me3 enriched block (Additional file 6C). In addition, and potentially as a consequence of hyperacetylation, both H3K27me3 and H3K9me3 signals were significantly decreased after incubation with HDACi. Taken together, our results demonstrate LBH-589-induced hyperacetylation (Additional file 6) not only in the whole nucleus, but also at the heterochromatic blocks of the X chromosomes.

### Induced hyperacetylation leads to prolonged substage and total S-phase duration

**Fig. 4:**
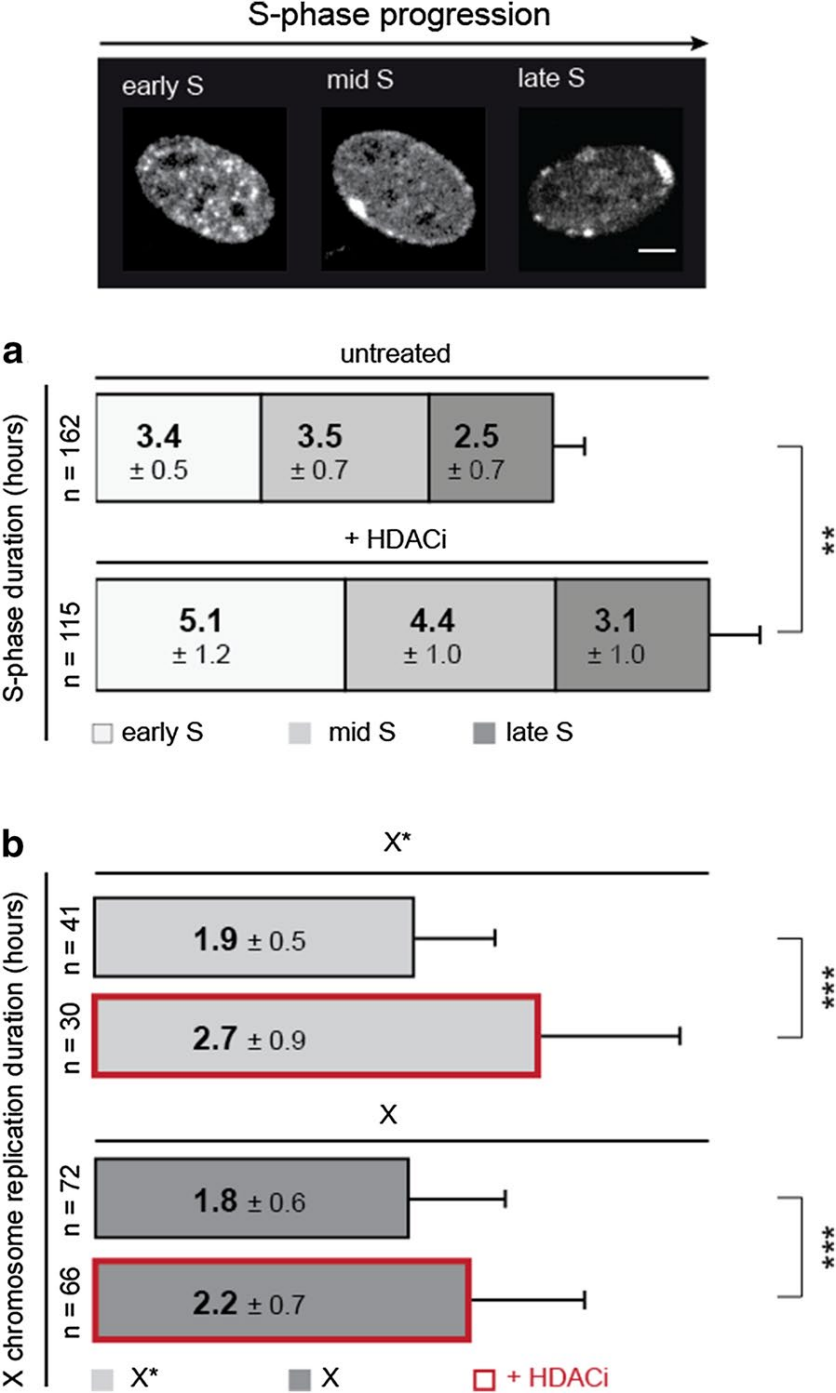
Hyperacetylation prolongs S-phase duration in total, substages and at sex chromosomes. **a** Exemplary images of S-patterns were depicted to illustrate the categorization into S-phase substages. *Microtus cabrerae* cells were transiently transfected with a plasmid encoding PCNA (either RFP or GFP-tagged) and either GFP-macroH2A1 or DsRed-HP1 beta. Cells were treated with DMSO or LBH-589 according to the protocol in the Additional file 4A and analyzed by live-cell imaging. In HDACi-treated cells, the total S-phase duration is significantly increased. Early S-phase duration increased from 3.4 up to 5.1 h, as well as mid-S-phase, which is prolonged by approximately 1 h. Sample sizes are indicated on the left-hand side. Statistical significance was tested using the *t* test, comparing the total S-phase duration and S-phase substage duration in untreated and treated samples. Standard deviations of replicates are shown in the boxes. **P < 0.01. **b** The duration of X chromosome replication was estimated out of live-cell imaging data. The duration of H3K27me3 decorated X* and H3K9me3 decorated X replication is significantly increased. The length of X* replication is prolonged from 1.9 up to 2.7 h and the length of X duplication from 1.8 h up to 2.2 h. Standard deviations of replicates are shown next to the numbers in the boxes. Statistical significance was tested using the *t* test, comparing the duration of replication of X* and X in untreated and treated samples. ***P < 0.001

After setting up an experimental approach to manipulate the histone acetylation level of the heterochromatic blocks, we next asked whether this global hyperacetylation affects the length of total S-phase, substages as well as the duration of sex chromosome replication. To test this, we first analyzed images from live-cell imaging experiments of several hours in 20 min intervals to unequivocally distinguish between the three different substages of S-phase (Fig. 4a). Based on these live-cell data, we measured the total S-phase duration, substage durations and sex chromatin replication duration in untreated and treated *Microtus cabrerae* cells.

Total S-phase duration was significantly prolonged from 9.4 up to 12.6 h in treated cells. In addition, the duration of the different S-phase substages increased as well. For early S-phase, it changed from 3.4 h up to 5.1 h, for mid-S-phase from 3.5 h up to 4.4 h, and for late S-phase from 2.5 h up to 3.1 h. With the help of double transfections using constructs encoding RFP-PCNA in combination with GFP-macroH2A1 or GFP-PCNA together with DsRed-HP1 beta constructs, we further calculated the duration of DNA replication at the heterochromatic blocks of the X chromosomes (Fig. 4b). Our analyses demonstrated that not only the total S-phase duration and the substages were affected by the global hyperacetylation, but also the duration of DNA replication of the heterochromatic blocks was prolonged. While the heterochromatic blocks were both replicated in a time frame of 1.8–1.9 h in control cells, after HDACi treatment, the duration of the H3K27me3/macroH2A-enriched heterochromatin replication increased up to 2.7 h and the duration of H3K9me3/HP1 beta-enriched heterochromatin replication up to 2.2 h. This increase in S-phase duration might be a consequence of delayed CDK-cyclin kinase activation due to an induction of cyclin-dependent kinase inhibitors [28]. Further studies are necessary to delineate whether this delayed S-phase progression is causally related to the DNA damage seen in HDACi-treated cells [29, 30].

### Site-directed targeting of histone acetyltransferase increases histone acetylation level and prolongs DNA replication duration of the constitutive heterochromatin

**Fig. 5:**
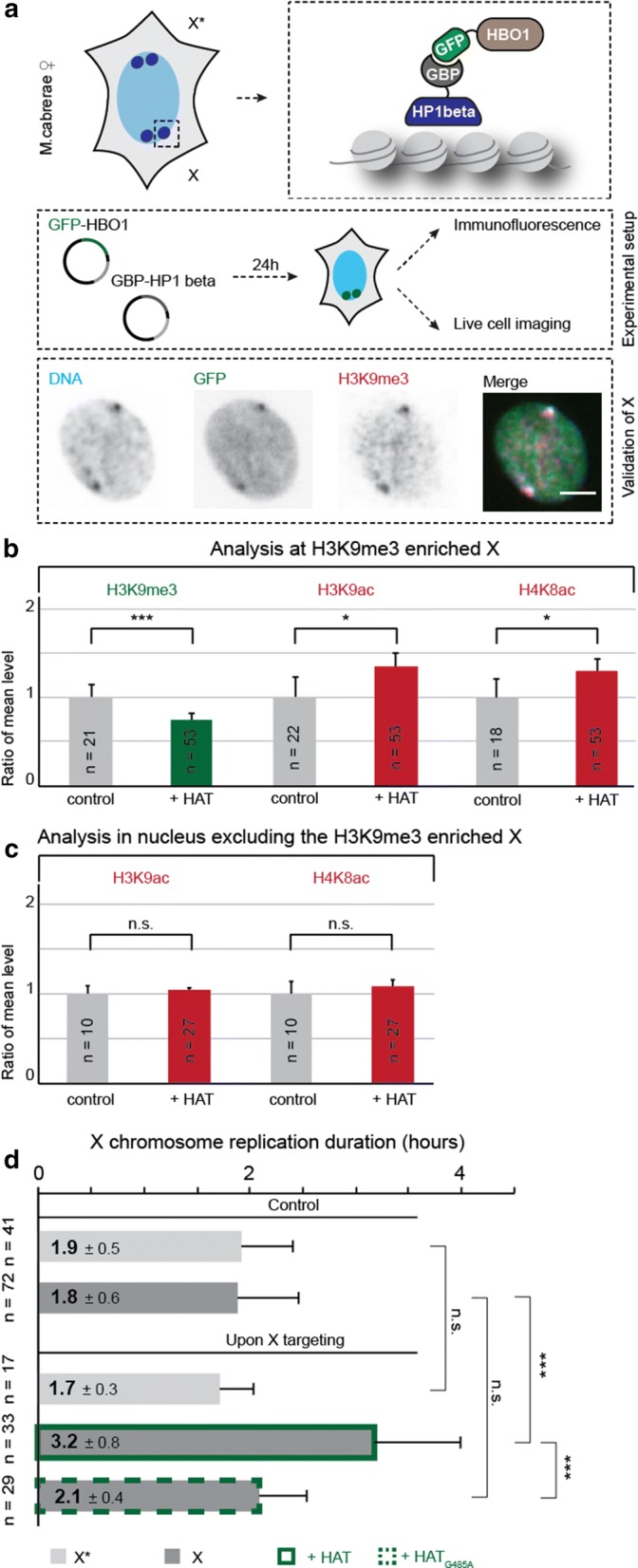
Specific targeting of histone acetyltransferase leads to hyperacetylation and increase in DNA replication duration of constitutive heterochromatin. **a** Schematic representation of the targeting approach in a *Microtus cabrerae* cell. HBO1, a histone acetyltransferase, was tagged to GFP, and HP1-beta was tagged to GBP, a GFP-binding protein. Upon co-expression of both and because of their strong interaction, HBO1 is specifically targeted to the H3K9me3 decorated X chromosome. The experimental setup (middle box) implied the transient transfection of two plasmids: GFP-HBO1 and GBP-HP1-beta, followed by an incubation time of 24 h. The functionality of the targeting was validated by antibody detection of H3K9me3 a marker of constitutive heterochromatin, resulting in a strong colocalization of DAPI-stained DNA in blue, GFP-HBO1 and GBP-HP1-beta in green and H3K9me3 in red. The merge shows an overlay of all three channels. Scale bar = 5 µm. **b** Untargeted and targeted cells were analyzed with a user-independent analysis to measure H3K9me3 and acetylation level at the X chromosome. Bar graphs indicate the ratio of the mean levels, where grayish bars represent the normalized control and colored bars the respective targeted sample. Statistical significance was tested using the *t* test, comparing untargeted and targeted cells. Error bars demonstrate 95 Cl. ***P < 0.001. **c** Untargeted and targeted cells were analyzed with a user-independent analysis to measure H3K9me3 and acetylation levels in the whole nucleus excluding the H3K9me3 decorated X chromosome. Bar graphs indicate the ratio of the mean levels, where grayish bars represent the normalized control and colored bars the respective targeted sample. Statistical significance was tested using the *t* test, comparing untargeted and targeted cells. Error bars demonstrate 95 Cl. **d** The duration of X chromosome replication was estimated out of live-cell imaging data. Error bars demonstrate standard deviation. Statistical significance was tested using the *t* test, comparing the duration of replication of X* and X in untargeted and targeted samples. As a negative control, a catalytically dead HBO point mutant (G485A) was used. ***P < 0.001

Together with an effect of global hyperacetylation on replication dynamics, we also detected a decrease in histone methylation marks in cells after treatment with HDACi (Fig. 3, Additional file 4), which could themselves have a direct impact on the DNA replication dynamics. To circumvent global effects, we next set up a targeting approach to specifically target HBO1, a histone acetyltransferase (HAT), to the X chromosome heterochromatic block enriched in H3K9me3 and HP1 beta (Fig. 5). Our targeting system consisted of two fusion proteins: GFP-tagged histone acetyl transferase (HBO1) and a GFP-binding protein (GBP) linked to HP1 beta that allows the recognition of the constitutive heterochromatin of the X chromosome. In a targeted state, HP1 beta binds to the constitutive heterochromatin of the X chromosome, while its GBP domain interacts with GFP-HBO1. This interaction resulted in the successful recruitment of HBO1 to the constitutive heterochromatin (Fig. 5a). To validate the successful site-directed targeting approach, we stained for H3K9me3 as a hallmark of the constitutive heterochromatin. Indeed, we detected a strong colocalization of the targeting signal and H3K9me3.

As a next step, we elucidated the effect of HBO1 targeting on the H3K9me3 level and histone acetylation level (H3K9ac and H4K8ac) at the HP1-enriched heterochromatic blocks (Fig. 5b). HBO1 targeting leads to a significant decrease in H3K9 trimethylation signal and a significant increase in the acetylation (Fig. 5b). To validate the specificity of the targeting, we performed a second analysis and created a binary mask, excluding the heterochromatic block signal and measuring thus only the remaining part of the nucleus (Additional file 5). Using this mask, we observed that histone acetylation and histone methylation levels remained unaltered, validating that this site-directed targeting approach specifically modulated the acetylation levels of the constitutive heterochromatin (Fig. 5c). Next, we elucidated the impact of site-directed targeting of HBO1 on the DNA replication duration of the constitutive heterochromatin. For that, we transfected cells with constructs encoding RFP-PCNA, to visualize active replication sites, in combination with GFP-HBO1 and GBP-HP1 beta, to allow HAT targeting. In addition, we performed the same experiment replacing the catalytic active HBO1 by a point mutant rendering HBO1 catalytically dead (HBO1-G485A). Using time-lapse microscopy, we elucidated the duration of replication for the heterochromatic blocks of the X chromosomes as described above. We observed a significant prolongation of the DNA replication duration of the H3K9me3/HP1-enriched heterochromatic block of the X chromosome when active HBO1 was targeted, which increase from 1.8 h up to 3.2 h. This was not the case when targeting the HBO1 catalytic mutant (Fig. 5d). Moreover, the replication timing of the other, H3K27me3/macroH2A enriched, heterochromatin block of the X chromosome remained unaltered, as a result of the specific targeted increase in histone acetylation level only at the H3K9me3-enriched heterochromatin block (Fig. 5d). With this additional approach of HP1-mediated targeting of HBO1, the effect of hyperacetylation on DNA replication timing was more dramatic than the global drug-induced hyperacetylation approach. Although HBO1 is known as a H4-specific histone acetyltransferase, we not only observed an effect on H4K8ac, but also on H3K9ac, indicating an interaction between HBO1 and histone H3. Studies have shown an interaction between HBO1 and H3K14ac [31], indicating potential interactions also with histone H3. Upon HAT targeting, we also achieved a loss of H3K9 trimethylation mark, similar to our results by global hyperacetylation. Previous studies have shown that a loss of H3K9me3 was not sufficient to change replication timing of constitutive heterochromatin [9]. Although we cannot exclude an additional effect of H3K9me3, altogether the data suggest that the level of histone acetylation at a given genomic region is a major factor in determining its DNA replication kinetics.

Furthermore, we performed HAT targeting also to the H3K27me3/macroH2A enriched X chromosome, using GBP-macroH2A1 and GFP-HBO1. This targeting approach was less effective, possibly due to the required incorporation of ectopic macroH2A1 into endogenous nucleosomes, which should take place with very slow kinetics, whereas HP1 beta simply needs to bind to H3K9 trimethylated nucleosomes and its exchange rate is relatively fast. Nevertheless, we were able to detect a prolongation of DNA replication of the facultative heterochromatic block at the X chromosome upon HAT targeting (Additional file 7). In line with our previous finding, there was no effect on the H3K9me3/HP1-enriched heterochromatic block of the X chromosome, further underlining the specificity of this site-directed targeting approach.

### Slower nucleotide incorporation rate in hyperacetylated *Microtus cabrerae* cells

**Fig. 6:**
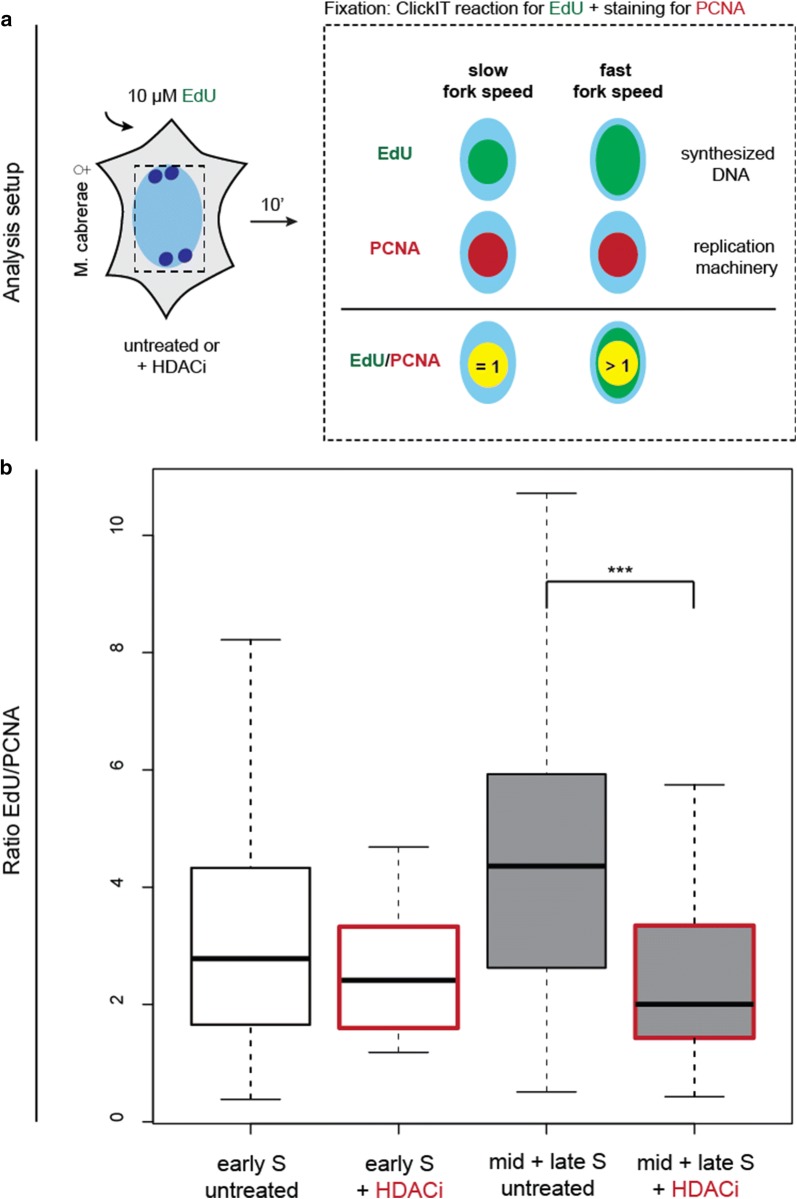
Induced hyperacetylation leads to a decrease in nucleotide incorporation rate and a slower fork speed. **a** Schematic representation of the calculation of relative nucleotide incorporation rate. Modified thymidine analog EdU was added for 10 min to *Microtus cabrerae* cells that were treated or untreated with HDACi prior to fixation. EdU was detected with ClickIT chemistry and endogenous PCNA via antibody detection. Whereas EdU represents the amount of synthesized DNA (incorporated nucleotides), PCNA reflects the replication machinery and thus active forks. For the estimation of nucleotide incorporation rate, the ratio of EdU (incorporated nucleotides) and PCNA (replication machinery) was estimated, as a marker for the speed of replication forks. If the ratio shows a value = 1, this means a complete overlap of both signals (EdU and PCNA) and indicates a slow replication fork speed and thus slower replication forks. If the ratio of both signals is > 1, this means that there was more DNA synthesized, indicating faster replication forks and hence a faster fork speed. **b** The ratio of EdU and PCNA signals was plotted as box plots. Cells were categorized by visual inspection of the EdU signal into early (light gray box) and mid + late cells (dark gray box). HDACi-treated samples are indicated by the red-framed box. In untreated cells, the nucleotide incorporation rate increases over time, demonstrating an increase in fork speed from early to mid + late cells. In contrast to untreated cells, HDACi-treated samples show a significantly lower ratio of EdU/PCNA signal, indicating a slower nucleotide incorporation rate and thus a slower fork speed. Statistical significance was tested using the Wilcoxon test, comparing untreated and HDACi-treated *Microtus cabrerae* cells. ***P < 0.001

We next asked whether the strong increase in the duration of total S-phase, its substages and on the length of replication of the heterochromatic blocks in response to hyperacetylation was a consequence of slower fork speed, and thus, more time was required to replicate the genome. To answer this question, we produced global inhibition of HDACs by incubation with LBH-589 and analyzed its effect on DNA replication fork speed in vole *Microtus cabrerae* cells, to understand the mechanism behind the prolongation of DNA replication. Thus, cells were treated with HDACi for 24 h, and afterward modified nucleotides were allowed to be incorporated for 10 min before fixing and staining for nucleotides and PCNA. We analyzed the fork speed of untreated and HDACi-treated samples with the help of total signal intensities and ratiometric analysis of nucleotide incorporation rate (Fig. 6a, Additional file 8). While PCNA is part of the DNA replication machinery and is therefore proportional to the numbers of active replisomes, the amount of incorporated nucleotides is proportional to both the number of active replisomes and the replication fork speed. By calculating the ratio of the total nucleotide signal to the total PCNA signal, we assessed changes in the relative replication fork speed in treated and untreated samples. As the normalized ratio of EdU/PCNA become higher, this indicates more synthesized DNA per active replisomes and consequently faster replication forks (Fig. 6a, Additional file 8). We plotted the nucleotide incorporation rate as box plots over S-phase progression from early S to mid- and late S-phase (Fig. 6b). In untreated samples, the nucleotide incorporation rate clearly increased over time approximately 1.4 times from early S to mid- and late S. This nucleotide incorporation rate increase is similar to our previous measurements in human cells [32]. In HDACi-treated samples there was, however, no increase in the replication fork speed along S-phase progression. When comparing early S-phase in treated versus untreated samples, we detected a slight decrease in nucleotide incorporation rate about 0.9 times. However, when comparing mid-S and late S in untreated versus HDACi-treated samples, the fold change was much greater decreasing to 0.6 of the synthesis rate in untreated cells. Our results indicate that hyperacetylation produces a significant decrease in nucleotide incorporation rate and, consequently, slower fork speed upon mid-S-phase progression in *Microtus cabrerae* cells.

### Hyperacetylated cells replicate more DNA during early S-phase and have decreased genomic duplication rates

**Fig. 7:**
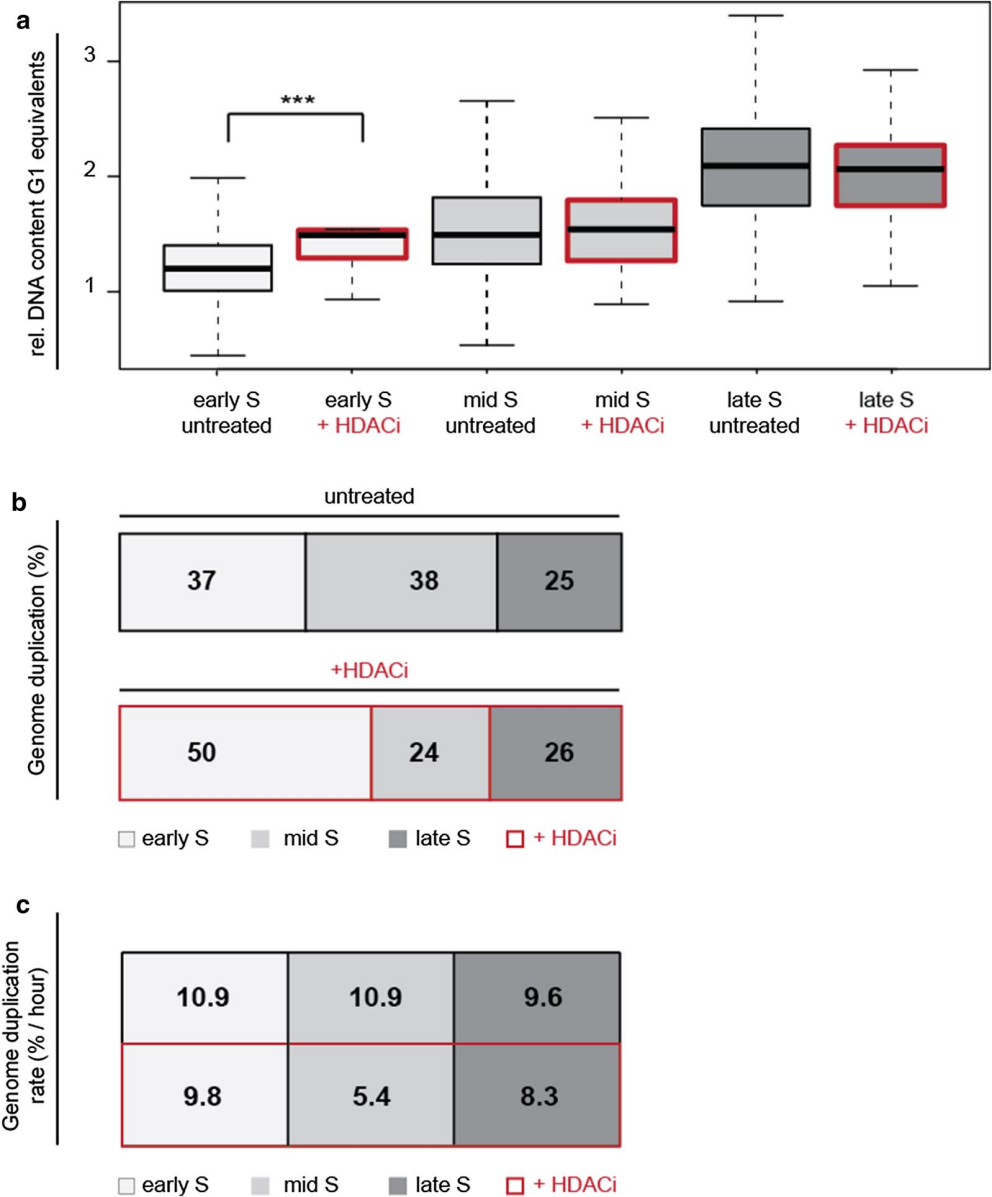
Hyperacetylated *Microtus cabrerae* cells show a stronger increase in genomic DNA in early S-phase and a decrease in the genome duplication rate. **a** The DNA content frequency analysis was performed by DAPI intensity measurements in treated and untreated cells, which were categorized into their respective S-phase substage according to their replication pattern. Box plots depict S-phase substages from early S to late S for both untreated and treated samples. HDACi-treated samples are indicated by the red-framed box. The DNA content of treated cells is in early S-phase significantly increased in comparison with untreated cells. Statistical significance was tested using the Wilcoxon test, comparing untreated and HDACi-treated *Microtus cabrerae* cells. ***P < 0.001. **b** The genome duplication time was calculated from the DNA content data. As after S-phase 100% of the DNA is replicated, the genome duplication per substage can be estimated. In treated cells (red-framed box), 50% of the genome was already replicated during early S-phase, whereas only 37% of the genome was replicated in early S in control samples. 38% of the genome was replicated in mid-S-phase in control cells. In contrast, only 24% of the genome was replicated in mid-S-phase in HDACi-treated cells. **c** When combining these genome duplication data with the measured DNA replication substage durations of Fig. 4a, we were able to measure the % of genome increase per hour, an indicator of replication speed. The values showed that the % of genome replicated in 1 h was decreased in treated samples, in particular, of facultative heterochromatin

Next, we wanted to address the question of whether the onset of DNA replication was affected by the histone hyperacetylation induced upon HDAC inhibition in *Microtus cabrerae* cells. As euchromatin is hyperacetylated and known to replicate during early S-phase, we tested whether upon induced genome wide hyperacetylation the replication timing of the heterochromatin also starts prematurely. To test this hypothesis, we plotted the relative DNA content measured as G1 equivalents from early S to late S-phase. For this purpose, thymidine analogs were allowed to be incorporated in *Microtus cabrerae* cells for 10 min and cells were fixed and stained. We visually categorized replicating cells according to the replication signal (EdU) in early, mid- and late S-phase as well as in non-S-phase cells (Fig. 7a). We analyzed the integrated DAPI intensity in individual nuclei and normalized all cells of one replicate to the G1 peak intensity as previously described [32]. As expected, upon DNA replication during S-phase the DNA content increases over time in untreated cells. We found that the amount of genomic DNA was significantly increased only during early S-phase in hyperacetylated cells compared to untreated cells, while in mid- and late S-phase no increment was observed. In addition, we validated these data in a manner independent of replication pattern classification, using genomic DNA increase during the S-phase (Additional file 9). Furthermore, we estimated the fraction of genome duplicating in untreated and HDACi-treated cells in every S-phase substage (Fig. 7b). In line with our previous outcome, we observed that 50% of the genome in hyperacetylated cells was already replicated in early S-phase, whereas in untreated cells only 37% of the genome was replicated during early S-phase. By contrast, during mid-S-phase the fraction of genome replicating was decreased to 24% in HDACi-treated cells, while in untreated cells it was 38%. During late S-phase, the fraction of genome duplicating remained similar in both treated and untreated cells. Finally, we combined these findings from the genome duplication rate analysis with data from live-cell imaging experiments on substage durations to calculate the percentage of the genome replicated per hour as an indicator of genome duplication speed (Fig. 7c). This analysis matched the data from the nucleotide incorporation rate (Fig. 6b) indicating that the replication speed was reduced upon HDACi treatment. Importantly, the genome duplication rate was dramatically altered in particular during mid-S-phase when it was approximately twofold reduced, while in the other S-phases substages the reduction was less pronounced.

**Fig. 8:**
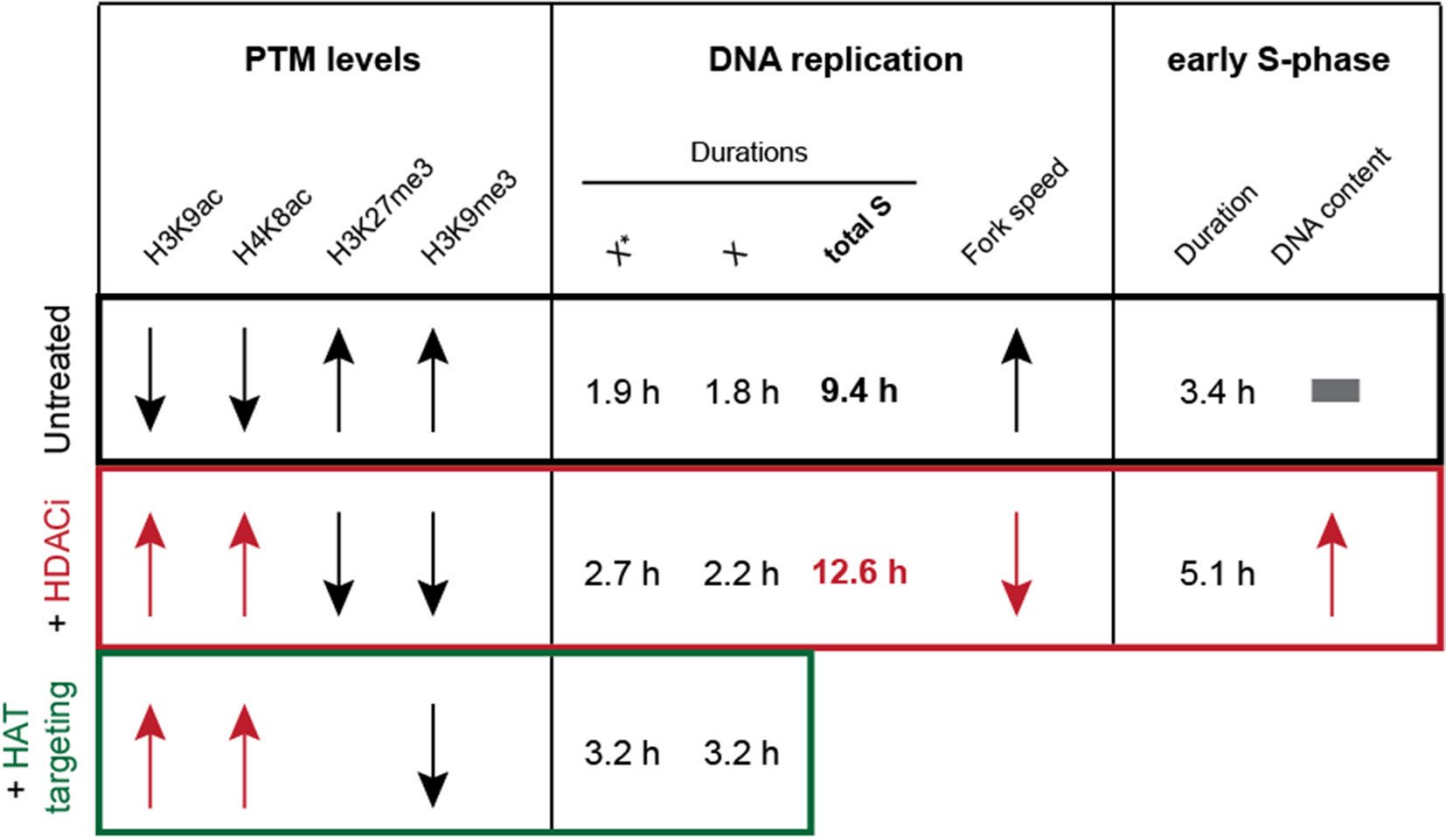
Summary of the effects of histone hyperacetylation on DNA replication timing. In untreated cells, (black box) showed typical marks for heterochromatin. Both heterochromatic blocks were hypoacetylated, but enriched for either H3K27me3 (X*, facultative heterochromatin) or for H3K9me3 (X, constitutive heterochromatin). These cells replicate in 9.4 h, where 1.9 h was required for the DNA replication of the H3K27me3 decorated X chromosome and 1.8 h for the duplication of the H3K9me3 decorated X chromosome. When cells were globally hyperacetylated with an HDAC inhibitor (red box), the histone acetylation level increases, but histone methylation marks decreased. The effect on DNA replication timing was dramatic, as the total S-phase duration was prolonged up to 12.6 h. The heterochromatic block of the X* was replicated in a time frame of 2.7 h, and constitutive heterochromatin of the X required 2.2 h for DNA duplication. This result, going hand in hand with a strong increase in DNA content in early S-phase, indicated a shift from facultative heterochromatin, which is normally replicated during mid-S-phase, toward early S-phase. The duration of early S-phase was also prolonged as a result of slower fork speed in treated samples. When the constitutive heterochromatin was targeted with a HBO1 (green box), we achieved histone hyperacetylation specifically at the H3K9me3 decorated X and, again, a loss of histone methylation marks. Our HAT targeting data confirmed the effect of histone hyperacetylation on DNA replication timing of genomic regions, as we also observed a prolongation of constitutive heterochromatin replication. That this approach is site specific was demonstrated by the fact that the DNA replication of the H3K27me3 decorated X chromosome was not affected by the targeting of HBO1 to the HP1 beta containing heterochromatin

All in all, our results, summarized in Fig. 8, demonstrated that histone acetylation level is a regulator of DNA replication timing of the *Microtus cabrerae* genome. Upon induction of hyperacetylation, irrespective of global induction by drug treatment or by specific HAT targeting, we observed a global increase in DNA replication duration (Figs. 4, 5). Therefore, histone acetylation levels negatively regulate replication fork speed at a global level in these cells. As the mean H4K8 acetylation level of the heterochromatic block of the H3K27me3/macroH2A-enriched X chromosome was higher in comparison with the H3K9me3/HP1 beta-enriched X chromosome (Additional file 6C), we conclude that higher acetylation level thereof leads to the shift from mid-to-early S-phase. Furthermore, the general prolongation of DNA replication suggests that the total number of active origins at any given time point remains constant in accordance to a limiting factor model (reviewed in [33]) and that there are not more origins firing to counterbalance the reduced fork speed. Ultimately, DNA replication timing is defined by the timing when DNA replication origins fire in addition to replication fork rate. The relative efficiency model of origin firing claims early origins to fire more efficiently, whereas late origins exhibit a lower efficiency at the beginning of S-phase, increasing over time when S-phase progresses, assuring the closure of potential gaps of unreplicated DNA in a timely fashion [34]. Several processes that lead to origin firing itself are known at which histone acetylation might regulate replication timing. In fission yeast, the dynamic of origin firing could be a result of differences in the timing of origin recognition complex (ORC) binding at specific genomic regions [35]. The binding of limiting ORC factors is probably facilitated at acetylated and open chromatin. Another potential process being influenced and promoted by histone acetylation is the origin licensing, as HBO1-mediated histone acetylation in yeast has been shown to play a role in loading Mcm 2–7 complex [36], which itself is required for origin licensing [37]. The induction of hyperacetylation may promote origin licensing or is involved in the actual firing process by increasing accessibility by opening chromatin or by an increase in binding affinity to limiting factors such as Cdc45, respectively [20, 38], which has been shown to increase the firing efficiency of inefficient origins [35]. In addition, replication duration depends on fork rate. Using knockdown systems and inhibitors of HDACs, it was shown that hyperacetylation leads to a reduction in replication velocity and in an increase in replication stress [39], further underlining our results that upon induced hyperacetylation DNA replication duration is prolonged. Our study also indicates that histone hypoacetylation plays a major role in defining the late DNA replication timing of the heterochromatin of the X chromosome in female *Microtus cabrerae* cells. We observed that upon hyperacetylation, the amount of histone methylation marks—both H3K9me3 and H3K27me3—on the heterochromatic blocks decreased. Upon these conditions, replication onset is however only altered for H3K27me3-enriched heterochromatin, which shifted from mid-to-early S-phase, whereas H3K9me3-enriched heterochromatin followed its conventional late replicating pattern. Altogether, the histone methylation signal appeared not as primary determinant of the replication timing of heterochromatin, which seemed to be directly modulated by histone acetylation levels. Our study, thus, highlights the occurrence of differences in the molecular mechanisms controlling the replication timing of the heterochromatic blocks at the differently epigenetically marked X chromosomes of *Microtus cabrerae* cells but also highlights a similar role of histone acetylation on replication kinetics across mammalian species.

## Conclusions

Our data reveal an impact of histone acetylation level on DNA replication timing in *Microtus cabrera*e cells. First, we demonstrated that the heterochromatic blocks coupled to the X chromosomes of female *Microtus cabrerae* cells showed typical marks for either facultative or constitutive heterochromatin. Moreover, we identified three distinct DNA replication patterns: early, mid and late in female *Microtus cabrerae* cells. The H3K27me3/macroH2A-enriched X chromosome-associated heterochromatic block was replicated during a narrow time frame of mid-S-phase, prior to the H3K9me3/HP1 beta-enriched X chromosome-associated heterochromatic block, which was replicated during late S-phase in concordance with their respective histone posttranslational pattern. Our data also suggest histone acetylation as a major determinant of DNA replication timing, as induced global as well as site-directed histone hyperacetylation leads to prolongation of total S-phase and sex chromosome replication duration. Furthermore, we detected a slower fork speed, when chromatin was hyperacetylated going hand in hand with an increase in DNA content replicated during early S-phase, indicating a shift from mid-replicating facultative heterochromatin toward early S-phase, when euchromatin was replicated. All in all, we highlighted a conserved role of histone acetylation on replication dynamics across mammalian species.

## Methods

### Expression plasmids

Expression vectors (Additional file 3) encoding human PCNA were either tagged to CFP (pc922, [40], GFP (pc595, [2]) or to mRFP (pc1054, [41] to visualize active replication sites. For the detection of the facultative heterochromatic block of the X chromosome, EGFP-tagged macroH2A1 (pc2101) was constructed with cDNA of human cells, amplified by PCR (macroH2A1-forward **EcoR1**: 5′-AA**GAATTC**AATGTCGAGCCGCGGTGGG; macroH2A1-reverse **Not1**: 5′-AA**GCGGCCGC**TAGTTGGCGTCCAGCTTGG) and cloned into pEGFP-C1 (Clontech). DsRed-HP1 beta was transfected to identify the constitutive heterochromatic block of the X chromosome (pc1225, [42]). For site-directed targeting of HBO1 to either of the heterochromatic blocks, different plasmids were generated. A construct encoding human HBO1 [43] was fused to C-terminal GFP of EGFP-C1 (pc852) (Clontech). A catalytic dead point mutant of HBO1 (G485A) was generated from the EGFP fusion using the primer 5′-ATGCCTCAGTACATGAGACAGGCCTATGGCAAGATGCTTA and matching reverse complement sequence. The resulting construct (pc2201) was verified by sequencing. To detect the facultative heterochromatic blocks of the X chromosome, a plasmid encoding GBP-macroH2A1 (pc2883) was constructed. The amplification of GBP was performed via PCR from GBP-MaSat (pc2469) [44]: (GBP-forward **AgeI**: 5′-A**TACCGGTA**TGGCCGATGTGC; GBP-reverse **XhoI**: 5′-ATCA**CTCGAGA**TGAGGAGACG). GFP-macroH2A1 (pc2101) was used as a backbone and was cut with AgeI and XhoI to create the final plasmid GBP-macroH2A1 (pc2883). To detect the constitutive heterochromatic block of the X chromosome, a construct encoding GBP-HP1 beta (pc3357) was created. MacroH2A1 was removed from GBP-macroH2A1 (pc2883) by restriction with EcoRI and BamHI and replaced by HP1 beta from DsRed-HP1 beta (pc1225, [42]).

### Cell culture, transfection and HDAC inhibitor treatment

Female *Microtus cabrerae* cells [45] as well as male *Microtus cabrerae* cells [23] were cultured in Dulbecco’s Modified Eagle’s Medium (DMEM) supplemented with 10% fetal calf serum and 1 µM gentamicin in 5% CO_2_ atmosphere at 37 °C. As positive control for 5mC staining, the mouse embryonal fibroblast W8 line [46] was used and cultured under standard conditions as described above with the addition of 1% non-essential amino acids. Cells used for immunofluorescence experiments were plated and grown on gelatinized glass coverslips. Transient transfections of female and male *Microtus cabrerae* cells were performed using nucleofection (Amaxa NucleoFector II, Lonza Ltd., Basel, Switzerland) with 1 µg per plasmid. For HDAC inhibition treatment, 50 nM panobinostat (LBH-589, Selleckchem, Houston, USA, Cat #: S1030) in PBS was added 24 h after seeding cells or nucleofection to the culture medium and incubated for at least 24 h prior to fixation or live-cell experiments (Additional file 4).

### Immunofluorescence and X chromosome FISH

Cells were grown on gelatinized glass coverslips, fixed in 4% paraformaldehyde (10 min at room temperature (RT)) and permeabilized for 20 min at RT in 0.5% Triton X-100/PBS. Immunofluorescence staining was performed in 4% BSA/PBS for 1 h at RT (primary antibodies) and in 4% BSA/PBS for 45 min at RT (secondary antibodies). The following primary antibodies were used: rabbit anti-H3K9ac (1/200, Upstate, Lake Placid, USA, Cat #: 06-942), rabbit anti-H4K8ac (1/200, Upstate, Lake Placid, USA, Cat #: 07-328) rabbit anti-H3K4me2 (1/800, Biomol, Hamburg, Germany, Cat #: BPS-25255), rabbit anti-H3K27me3 (1/200, Upstate, Lake Placid, USA, Cat #: 07-449) and rabbit anti-H3K9me3 (1/200, Upstate, Lake Placid, USA, Cat #: 07-442), mouse anti-H3K9me3 (1/100, Active Motif, Carlsbad, USA, Cat #: 39285), mouse anti-5mC (1/200, Active Motif, clone: 33D3). Secondary antibodies were goat anti-rabbit-IgG Alexa Fluor 488 (1/200, Invitrogen, Carlsbad, USA, Cat #: A-11008), donkey anti-mouse IgG-Cy3 (1/200, The Jackson Laboratory, Bar Harbour, USA, Cat #: 715-166-151), donkey anti-rabbit IgG-Cy5 (1/200, The Jackson Laboratory, Cat #: 711-175-152) or donkey anti-mouse IgG-Cy5 (1/200, The Jackson Laboratory, Cat #: 715-175-150). Nuclear DNA was visualized by 4,6-diamidino-2-phenylindole (DAPI, 1 µg/ml, Sigma Aldrich, Steinheim, Germany, Cat #: D9542). Cells were mounted in Vectashield antifade (Vector Laboratories, Burlingame, USA, Cat #: H-1000). For detection of 5mC modifications in nuclear DNA, the protocol described for 5hmC detection in Ludwig et al. [47] was used. Mitotic preparations and FISH analyses in *Microtus cabrerae* cell cultures followed standard protocols previously described earlier [24]. A painting probe from the X chromosome was prepared by micro-dissection combined with DOP-PCR labeling method (Fig. 1a). For immunodetection of histone modifications on metaphase chromosomes, the procedure described in Terrenoire et al. [48] was modified as followed: *Microtus cabrerae* cells were incubated for 3 h in 0.1 µg/ml colcemid (Roche). Mitotic cells were enriched by shake-off and collected by centrifugation (200 g, 5 min). Cells were hypotonically treated for 20 min at room temperature at a density of 1x10^5^ cells/ml 0.75 mM KCl. Cells were cyto-spun onto glass slides for 10 min at 400 g. Spread chromosomes were immediately fixed in 3.7% formaldehyde/PBS for 15 min, before cells were incubated in KCM buffer (120 mM KCl, 20 mM NaCL, 10 mM Tris/HCl pH 8.0, 0.5 mM EDTA, 0.1% Triton X-100) for 20 min. Subsequent immunodetection was performed as described above.

### In situ replication labeling

For the visualization of replicating DNA and epigenetic marks of heterochromatic blocks, female *Microtus cabrerae* cells were transiently transfected with CFP-PCNA and were pulse labeled for 20 min with 10 µM 5′-ethynyl-2′-deoxyuridine (EdU) (Invitrogen, Carlsbad, USA, Cat #: C10337) followed by a 1-h chase prior fixation with 4% paraformaldehyde. EdU was detected with the ClickIT system (Invitrogen) and AlexaFluor 488 followed by an immunostaining with rabbit anti-H3K27me3 (1/200, Upstate, Lake Placid, USA, Cat #: 07-449) and with mouse anti-H3K9me3 (1/100, Active Motif, Carlsbad, USA, Cat #: 39285). The following secondary antibodies were used: donkey anti-mouse IgG-Cy3 (1/200, The Jackson Laboratory, Bar Harbour, USA, Cat #: 715-166-151) and donkey anti-rabbit IgG-Cy5 (1/200, The Jackson Laboratory, Bar Harbor, USA, Cat #: 711-175-152). DNA was counterstained with 1 µg/ml DAPI for 10 min at RT, and cells were mounted afterward in Vectashield antifade (Vector Laboratories) (Fig. 2).

### In situ transcription labeling

Cells were incubated for 10 min in the presence of 1 mM EU (Thermo Fisher) to label nascent RNA [49]. Subsequently, cells were directly fixed in 3.7% formaldehyde/PBS for 15 min at RT. Incorporated EU was visualized using the ClickIT reaction with 6-FAM labeled azide (Carl Roth, Karlsruhe, Germany) according to the manufacturer’s recommendation (Thermo Fisher). Subsequently, X chromosome blocks were visualized by immunofluorescence staining with anti-H3K9me3 and anti-H3K27me3 antibodies as described above.

### Microscopy

Confocal images were obtained using an UltraVIEW VoX spinning disk system (PerkinElmer, Massachusetts, USA) on a Nikon Ti microscope equipped with an oil immersion Plan-Apochromat x60/1.45 NA objective lens (pixel size in XY = 112 µm, Z-step 0.3–1 µm). Excitation was done using the following laser lines: 405, 488, 561 and 640 nm. Images were taken with the appropriate filters for the respective dyes: DAPI: emission wavelength (em): 415–475 nm; GFP: em: 505–549 nm; RFP: em: 580–650 nm and Cy5: em: 664–754 nm. RGB stacks and montages were created using ImageJ (http://rsb.info.nih.gov/ij/). For live-cell microscopy, transfected female *Microtus cabrerae* cells were plated on a glass bottom p35 dish and were grown and HDACi treated under standard conditions. Time-lapse experiments were carried out in a closed live-cell microscopy chamber (ACU control, Olympus, Tokyo, Japan) heated to 37 °C, with 5% CO_2_ and 60% air humidity. Stacks were acquired at 20-min intervals and taken with a CCD camera. Immunofluorescence images of fixed cells were also captured with an Axiovert 200 microscope (Carl Zeiss, Jena, Germany) with a 63x/1.4 NA Plan-Achromatic oil objective lens (Carl Zeiss, pixel size in XY = 104 µm). Grayscale images were pseudocolored and merged using ImageJ. For nucleotide incorporation rate measurements and DNA content analysis, cells were imaged using the Operetta High Content imaging system (PerkinElmer, Massachusetts, USA), equipped with a 40×/0.95 NA air objective.

### Image analysis and quantification

For the quantification of histone acetylation levels (H3K9ac, H4K8ac), the mean values of acetylation were measured in the whole nucleus, at the sex chromosomes and in the whole nucleus excluding the X chromosomes (Figs. 3, 5, Additional files 4, 5, 7). Cells treated with HDACi were normalized to untreated samples. Quantification thereof was performed using a custom written software in the Python image analysis platform ((http://code.google.com/p/priithon/). Images were processed using a 3D median filter. Filtered images were threshold and then used to calculate the mean intensity of DAPI compaction, acetylation and methylation level. Analysis of total S, S substage and X*/X replication duration was performed by counting live-cell data time points and categorizing cells into early, mid- or late S-phase stage according to their PCNA pattern. An early S-phase pattern is characterized by DNA replication foci distributed homogenously throughout the nucleus with exclusion of the nucle(ol)ar periphery, whereas a mid-S-phase pattern is identifiable by more organized foci at the nucle(ol)ar periphery and the replication of the H3K27me3/macroH2A enriched X chromosome(s). Late S-phase is clearly recognizable due to the appearance of larger DNA replication foci of the H3K9me3/HP1 beta-enriched X chromosome(s) (Fig. 4) .

### Ratiometric analysis of nucleotide incorporation rate

Modified thymidine analogs (EdU) were given to the cells for 10 min prior to fixation. EdU was detected with the ClickIT system (Invitrogen) followed by an immunostaining with mouse anti-PCNA (1/100, Dako, Santa Clara, USA, Cat #: M0879) and detection with donkey anti-mouse IgG-Cy3 (1/200, The Jackson Laboratory, Bar Harbour, USA, Cat #: 715-166-151). DNA was counterstained with 1 µg/ml DAPI for 10 min at RT and cells were afterward mounted in Vectashield antifade (Vector Laboratories). Detection thereof and fluorescence microscopy allowed the identification of early, mid- and late DNA replication patterns. *Microtus cabrerae* cells were imaged using the Operetta High Content imaging system. Cell segmentation and quantification of nuclear intensities were performed using Harmony (PerkinElmer, Massachusetts, USA). Whereas EdU shows the synthesized DNA (incorporated nucleotide), PCNA represents the replication machinery and, thus, active replisomes. For the calculation of nucleotide incorporation rate, the ratio of EdU (incorporated nucleotides) to PCNA (replication machinery) was estimated, as a proxy for the fork speed (Fig. 6). If the ratio shows a value = 1, this means a complete overlap of both signals (EdU and PCNA) and indicates a slow replication fork speed and thus slower replication forks. The higher the ratio of the total intensity, the higher the amount of DNA synthesized per replisome within the pulse and so the faster the fork speed.

### DNA content analysis and genome duplication rate

*Microtus cabrerae* cells were imaged using the Operetta High Content imaging system (PerkinElmer, Massachusetts, USA). For imaging constant exposure times and appropriate filter sets (DAPI: ex: 360–400 nm; em: 410–480 nm; GFP: ex: 460–490 nm; em: 500–560 nm; RFP: ex: 560–580 nm; em: 590–640 nm) were used. Cell segmentation and quantification of nuclear intensities were performed using Harmony (PerkinElmer, Massachusetts, USA). Subsequently, cells were manually staged for early, mid- or late S-phase based on their PCNA pattern. Based on this classification, the integrated DNA intensity (DAPI) per cell nucleus was plotted for all cells from each replicate (Fig. 7a). Based on the histogram of all cells per replicate, the DAPI intensity of each cell was normalized to the corresponding G1 and G2 peaks obtained by density fitting. This allowed pooling of the three replicates. Next, the normalized DAPI intensity per nucleus was classified in the corresponding S-phase substages for untreated and HDACi-treated samples. To directly measure the amount of genomic DNA synthesized in each substage, we analyzed the maximal percentage of DAPI in each substage normalized to G1 and estimated how much DNA in total gets synthesized during the respective substage. When dividing this value by the duration of replication for each substage, we achieved the percentage of the synthesized genome per hour, indicating the speed of DNA replication (Fig. 7b, c). Alternatively, for a replication pattern-independent analysis, the total nuclear DAPI intensities (corresponding to the DNA content) were first used to generate a histogram with 18 bins ranging from G1 to G2. Next, we calculated the relative frequency of manually assigned DNA replication patterns in each bin, both for treated and untreated cells. Statistical data for the plots are summarized in the tables provided as Additional files 10 and 11.

## Additional files

**Additional file 1.** Subnuclear distribution of facultative and constitutive heterochromatin marks in female *Microtus cabrerae* fibroblasts and transcription at X chromosomes. (A) Prominent chromatin marks were analyzed by immunostaining: H3K4me3, H3K9ac, H4K8ac (euchromatin), HP1 alpha (constitutive heterochromatin), MBD (DNA methylation) and 5-methyl-cytosine (5mC). Since 5mC staining was barely detectable in *Microtus cabrerae* cells, 5mC staining is also shown in MEF W8 cells where prominent staining at pericentromeric heterochromatin blocks is visible. DAPI-stained DNA (blue), immunostaining (red) and merge of both channels are depicted. A ROI around one heterochromatic block was selected, and a line intensity plot analysis was performed. Red arrows point to characteristic hypoacetylation of heterochromatic blocks. Scale bar 5 µm. (B) Transcriptional activity of heterochromatic blocks was assessed by the colocalization of nascent transcripts (10 minutes pulse of 1 mM EU) with the H3K9me3 and H3K27me3 enriched heterochromatin blocks. Quantification of EU signal within the two respective volumes after 3D segmentation showed significant higher levels in H3K9me3 blocks (***P < 0.001, paired t-test).

**Additional file 2: Movie 1.** Time-lapse analysis of female *Microtus cabrerae* cells throughout S-phase. Cells were triple transfected with CFP-PCNA (red), GFP-macroH2A1 (green) and DsRed-HP1 beta (blue) and imaged every 20 minutes. For details, see Figure 2. Scale bar 5 μm.

**Additional file 3.** Relevant parts of expression constructs used in this study. Schematic representation of relevant features of plasmids used in this study. Plasmid collection number (pc…), structure of the plasmid and, on the right-hand side, the corresponding reference of the plasmids are shown. Drawings are not scaled.

**Additional file 4.** Manipulation of heterochromatic blocks’ constitution by HDAC inhibitor. (A) Schematic representation of the experimental setup to manipulate the heterochromatic blocks’ constitution of *Microtus cabrerae* cells by HDACi LBH-589. LBH-589 was expected to increase histone acetylation level and potentially lead to decondensation and a potential effect on DNA replication timing. Cells were either seeded or transfected with the corresponding constructs (GFP-macroH2A1/ GFP-HP1 beta, RFP-PCNA) and incubated for 24 hours. 50 nM LBH-589 was added to the medium, and cells were again incubated for 24 hours. Control cells were treated with DMSO only. Afterward, cells were either fixed and subjected to immunostaining or used for live-cell imaging with a spinning disk confocal microscope. (B) Untreated and HDACi-treated female *Microtus cabrerae* fibroblasts were analyzed with a user-independent analysis, and the histone acetylation level in the whole cell nucleus was measured. Bar graphs of the mean acetylation level (red bar) in untreated cells and HDACi-treated cells. Sample sizes are indicated in the bars. Gray bars demonstrate the normalized control. Statistical significance was tested using the t-test, comparing untreated and HDACi-treated cells. Error bars demonstrate 95 Cl. ***P < 0.001.

**Additional file 5.** Schematic rationale of single steps for mask generation used for quantification of nuclear PTM levels in untreated and treated/targeted cells. Confocal images were obtained using an UltraVIEW VoX spinning disk system (PerkinElmer, Massachusetts, USA) on a Nikon Ti microscope equipped with an oil immersion Plan-Apochromat x60/1.45 numeric aperture objective lens (pixel size in XY= 112 μm, Z-step 0.3 μm). For the calculation of mean DAPI and mean PTM intensities (H3K9ac, H4K8ac, H3K27me3, H3K9me3) in the whole nucleus, at the heterochromatic block at the X chromosomes or in the whole nucleus excluding the X, mid-nuclear sections of the DAPI and GFP channel were used to generate nuclear, X and exclusion masks, respectively. Images were processed using a median 3D filter and were threshold in four successive steps. For the generation of the binary masks, all pixels below the final threshold were set to 1, for both masks, respectively. Total PTM level values overlapping with the respective mask were calculated and divided by the total number of pixels corresponding to the area of measurement. To automate this analysis procedure, a routine was written in the programming language Python (https://code.google.com/archive/p/priithon/). Mean values were measured and normalized to either untreated or untargeted samples.

**Additional file 6.** Titration analysis of potential HDAC inhibitors. (A) Overview of different HDAC classes and corresponding HDAC inhibitors of each class. MS-275 only affects HDAC1, HDAC2 and HDAC3 of class I (orange), whereas TSA and LBH-589 inhibit HDACs of class I, II and IV (blue/red). (B) Titration analysis of histone hyperacetylation in response to different HDAC inhibitors in female *Microtus cabrerae* cells. TSA treatment was performed over three days (72 hours). Previous studies have shown that a concentration of 20 nM was sufficient to hyperacetylate very condensed constitutive heterochromatin in C2C12 mouse cells [9]. Here, we increased this concentration 5x, 10x and 20x and did not achieve significant hyperacetylation at heterochromatic sex chromatin of *Microtus cabrerae* cells. Over time and at higher concentrations of TSA, cells showed morphological changes and with 800 nM cells died. In contrast, treatment with LBH-589 leads to hyperacetylation at sex chromatin already after one day of treatment. We were able to achieve significant increase in histone acetylation levels at the sex chromatin. To ensure a sufficient and stable hyperacetylation in the absence of morphological changes, we treated the cells with 50 nM for one day. MS-275 data are not shown as cells either showed same levels as controls or died directly after treatment. Statistical significance was tested using the t-test, comparing untreated and HDACi-treated cells. Error bars demonstrate 95Cl. ***P < 0.001. (C) Effect of HDACi treatment on histone acetylation level of female *Microtus cabrerae* cells. Treatment with LBH-589 leads to significantly increased H3K9ac and H4K8 mean acetylation level at sex chromatin. Mean H4K8ac level at untreated samples is significantly higher at the X* in comparison with the X. Statistical significance was tested using the t-test, comparing HDACi-treated cells and untreated cells, as well as mean H4K8ac levels in untreated cells at X* versus X. Error bars demonstrate 95Cl. ***P < 0.001.

**Additional file 7.** HBO1 targeting to the heterochromatic block of the H3K27me3 decorated X chromosome leads to prolongation of its DNA replication duration. (A) Schematic representation of targeting assay to specifically target HBO1 to the heterochromatic block of the H3K27me3 enriched X chromosome. Cells were transiently transfected with GFP-tagged HBO1 and GBP-macroH2A1. Upon co-expression, both counterparts of the targeting assay strongly interact, leading to the targeting of HBO1. Targeting was validated by immunostaining with H3K27me3 antibody as a hallmark of facultative heterochromatin. DNA (blue), GFP-HBO1 (green), H3K27me3 (red) and merge as an overlay of all three channels. Scale bar 5 µm. (B) Site-directed targeting of HBO1 to the H3K27me3-enriched X chromosome leads to a significant prolongation of DNA replication duration of the X*. Specificity of the targeting approach was validated by the fact that there was no significant difference in H3K9me3 decorated X replication duration upon X* targeting.

**Additional file 8.** Demonstration of ratiometric analysis of nucleotide incorporation rate in *Microtus cabrerae* control cell. Modified nucleotides were added to proliferating populations of *Microtus cabrerae* cells and were incorporated for 10 minutes. Cells were fixed, and EdU was detected via ClickIT chemistry. PCNA was stained with an antibody. Exemplary images of an early S-, mid-S- and late S-phase cell are shown: EdU (green), PCNA (red) and merge of both channels. Line profiles depict the ratio of EdU (synthesized DNA, green) and PCNA (DNA replication machinery, red). Scale bar 5 µm.

**Additional file 9.** DNA content analysis independent of S-phase pattern staging. The data shown in Figure 7A were binned using the DNA content of the total cell population (A) to test for user-dependent S-phase pattern classification biases. Then, the frequency of the S-phase patterns assigned was plotted per DNA content bin. The early (red), mid (blue) and late (green) S-phase patterns corresponded well to the progression of genomic DNA content increase during S-phase in untreated cells (B). In contrast, in LBH-treated cells the distribution of the early replication pattern occurred within cells with a higher DNA content (red), while the late replication pattern appeared unaffected.

**Additional file 10.** Plot statistics of main figures. Overview of results and statistics of data implemented in the main figures.

**Additional file 11.** Plot statistics of Supplementary Figures. Overview of results and statistics of data implemented in the additional files.

## Abbreviations

X*: X chromosome with H3K27me3/macroH2A enriched heterochromatin block
X: X chromosome with H3K9me3/HP1 beta-enriched heterochromatin block
EdU: 5′-ethynyl-2′-deoxyuridine
PCNA: proliferating cell nuclear antigen
HDACi: histone deacetyltransferase inhibitor
HAT: histone acetyltransferase
LBH-589: panobinostat
*N*: sample size
