# Supplementary figures and images for: DNA replication dynamics of vole genome and its epigenetic regulation
Source: Epigenetics Chromatin. 2019 Mar 14;12:18. doi: 10.1186/s13072-019-0262-0 (PMC6416958; doi:10.1186/s13072-019-0262-0)

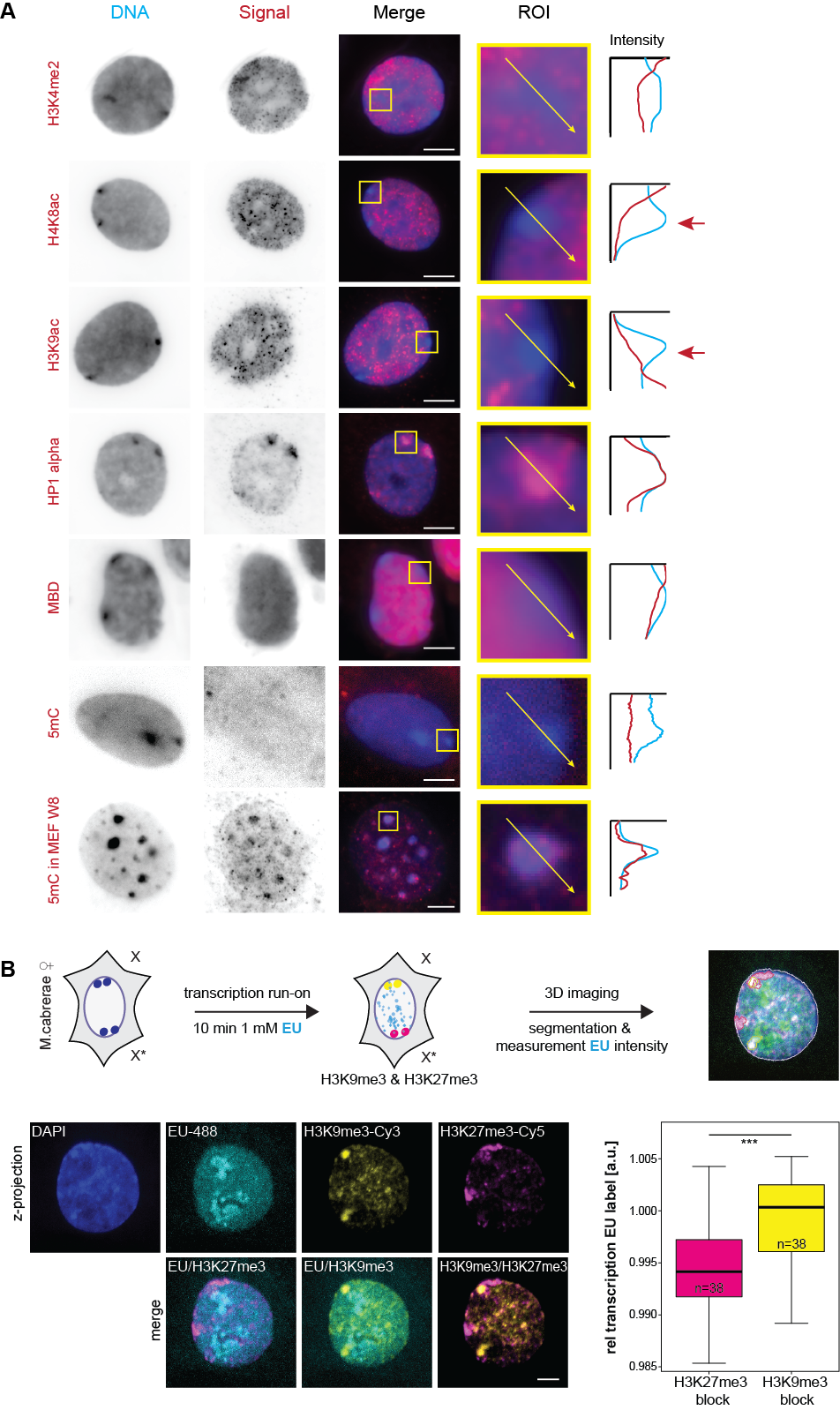

Supplement: Supplementary file 1 — Additional file 1. Subnuclear distribution of facultative and constitutive heterochromatin marks in female Microtus cabrerae fibroblasts and transcription at X chromosomes. (A) Prominent chromatin marks were analyzed by immunostaining: H3K4me3, H3K9ac, H4K8ac (euchromatin), HP1 alpha (constitutive heterochromatin), MBD (DNA methylation) and 5-methyl-cytosine (5mC). Since 5mC staining was barely detectable in Microtus cabrerae cells, 5mC staining is also shown in MEF W8 cells where prominent staining at pericentromeric heterochromatin blocks is visible. DAPI-stained DNA (blue), immunostaining (red) and merge of both channels are depicted. A ROI around one heterochromatic block was selected, and a line intensity plot analysis was performed. Red arrows point to characteristic hypoacetylation of heterochromatic blocks. Scale bar 5 µm. (B) Transcriptional activity of heterochromatic blocks was assessed by the colocalization of nascent transcripts (10 minutes pulse of 1 mM EU) with the H3K9me3 and H3K27me3 enriched heterochromatin blocks. Quantification of EU signal within the two respective volumes after 3D segmentation showed significant higher levels in H3K9me3 blocks (***P < 0.001, paired t-test). [file 13072_2019_262_MOESM1_ESM.png]

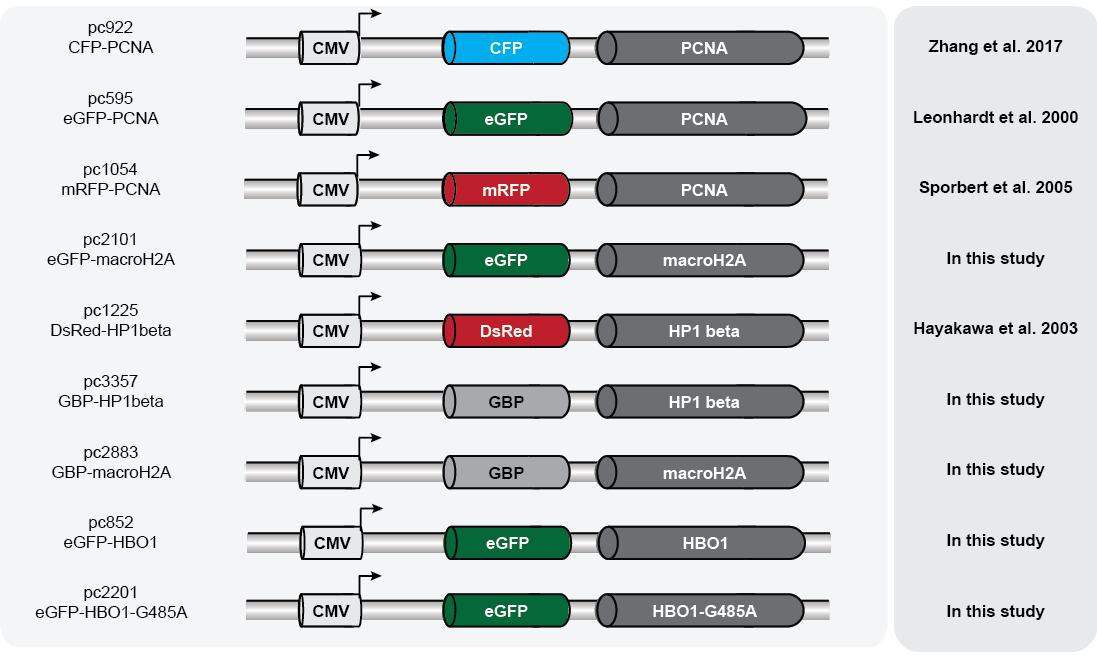

Supplement: Supplementary file 3 — Additional file 3. Relevant parts of expression constructs used in this study. Schematic representation of relevant features of plasmids used in this study. Plasmid collection number (pc…), structure of the plasmid and, on the right-hand side, the corresponding reference of the plasmids are shown. Drawings are not scaled. [file 13072_2019_262_MOESM3_ESM.png]

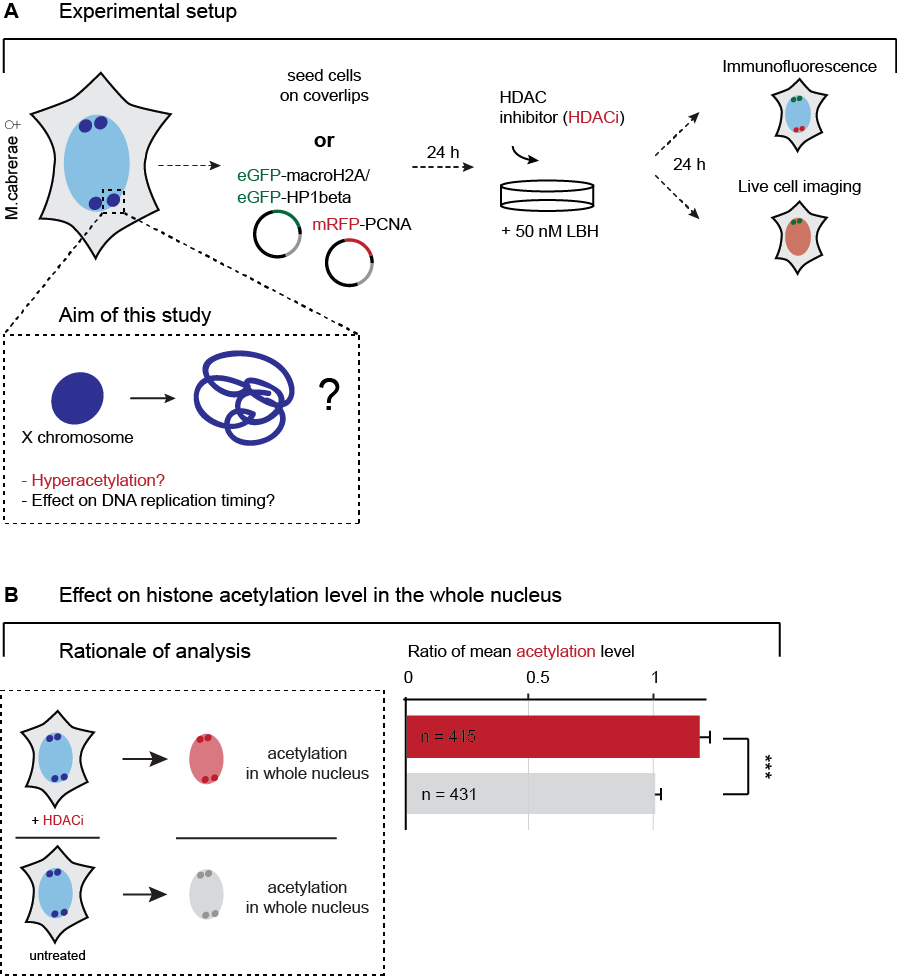

Supplement: Supplementary file 4 — Additional file 4. Manipulation of heterochromatic blocks’ constitution by HDAC inhibitor. (A) Schematic representation of the experimental setup to manipulate the heterochromatic blocks’ constitution of Microtus cabrerae cells by HDACi LBH-589. LBH-589 was expected to increase histone acetylation level and potentially lead to decondensation and a potential effect on DNA replication timing. Cells were either seeded or transfected with the corresponding constructs (GFP-macroH2A1/ GFP-HP1 beta, RFP-PCNA) and incubated for 24 hours. 50 nM LBH-589 was added to the medium, and cells were again incubated for 24 hours. Control cells were treated with DMSO only. Afterward, cells were either fixed and subjected to immunostaining or used for live-cell imaging with a spinning disk confocal microscope. (B) Untreated and HDACi-treated female Microtus cabrerae fibroblasts were analyzed with a user-independent analysis, and the histone acetylation level in the whole cell nucleus was measured. Bar graphs of the mean acetylation level (red bar) in untreated cells and HDACi-treated cells. Sample sizes are indicated in the bars. Gray bars demonstrate the normalized control. Statistical significance was tested using the t-test, comparing untreated and HDACi-treated cells. Error bars demonstrate 95 Cl. ***P < 0.001. [file 13072_2019_262_MOESM4_ESM.png]

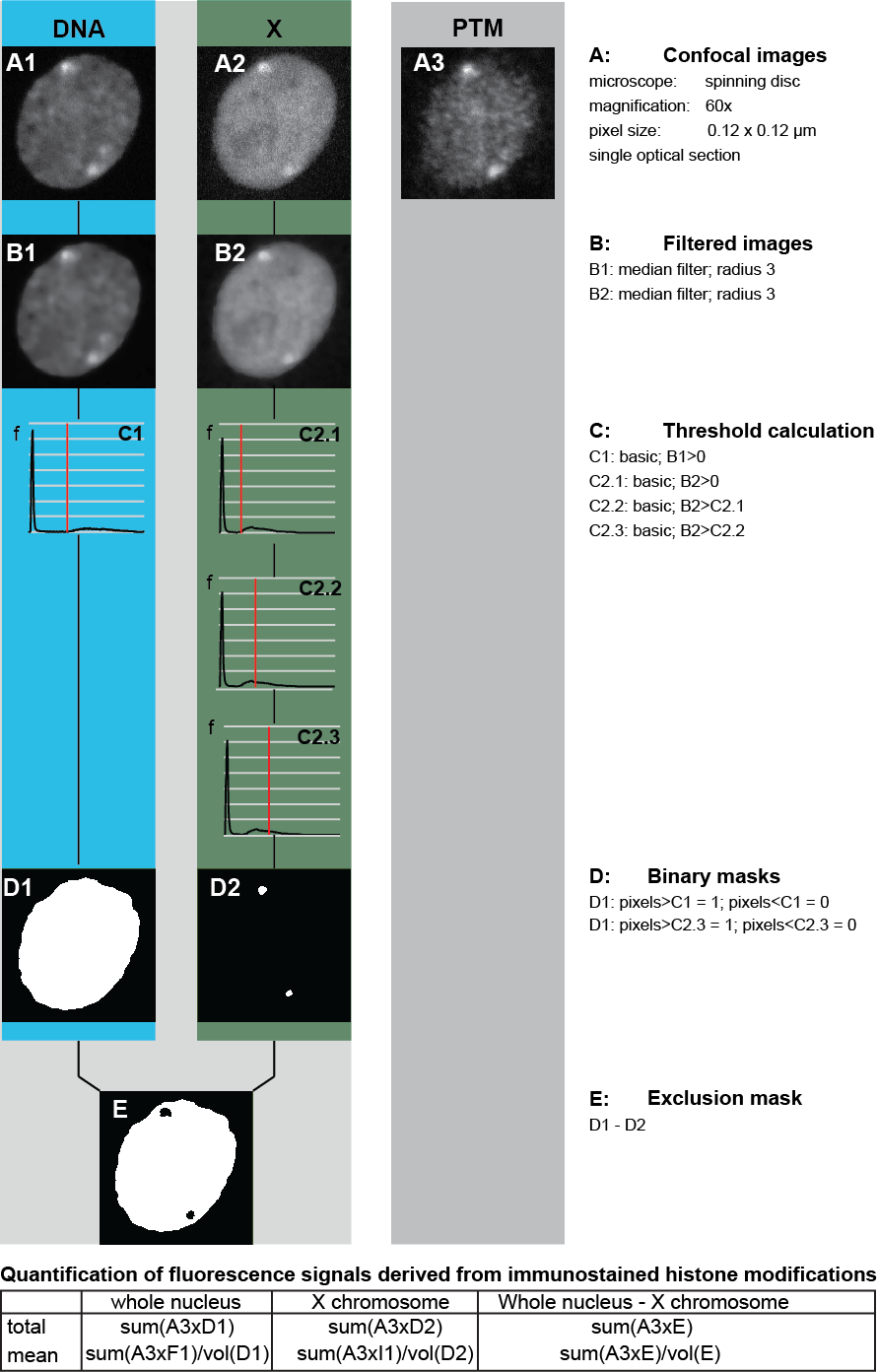

Supplement: Supplementary file 5 — Additional file 5. Schematic rationale of single steps for mask generation used for quantification of nuclear PTM levels in untreated and treated/targeted cells. Confocal images were obtained using an UltraVIEW VoX spinning disk system (PerkinElmer, Massachusetts, USA) on a Nikon Ti microscope equipped with an oil immersion Plan-Apochromat x60/1.45 numeric aperture objective lens (pixel size in XY= 112 μm, Z-step 0.3 μm). For the calculation of mean DAPI and mean PTM intensities (H3K9ac, H4K8ac, H3K27me3, H3K9me3) in the whole nucleus, at the heterochromatic block at the X chromosomes or in the whole nucleus excluding the X, mid-nuclear sections of the DAPI and GFP channel were used to generate nuclear, X and exclusion masks, respectively. Images were processed using a median 3D filter and were threshold in four successive steps. For the generation of the binary masks, all pixels below the final threshold were set to 1, for both masks, respectively. Total PTM level values overlapping with the respective mask were calculated and divided by the total number of pixels corresponding to the area of measurement. To automate this analysis procedure, a routine was written in the programming language Python (https://code.google.com/archive/p/priithon/). Mean values were measured and normalized to either untreated or untargeted samples. [file 13072_2019_262_MOESM5_ESM.png]

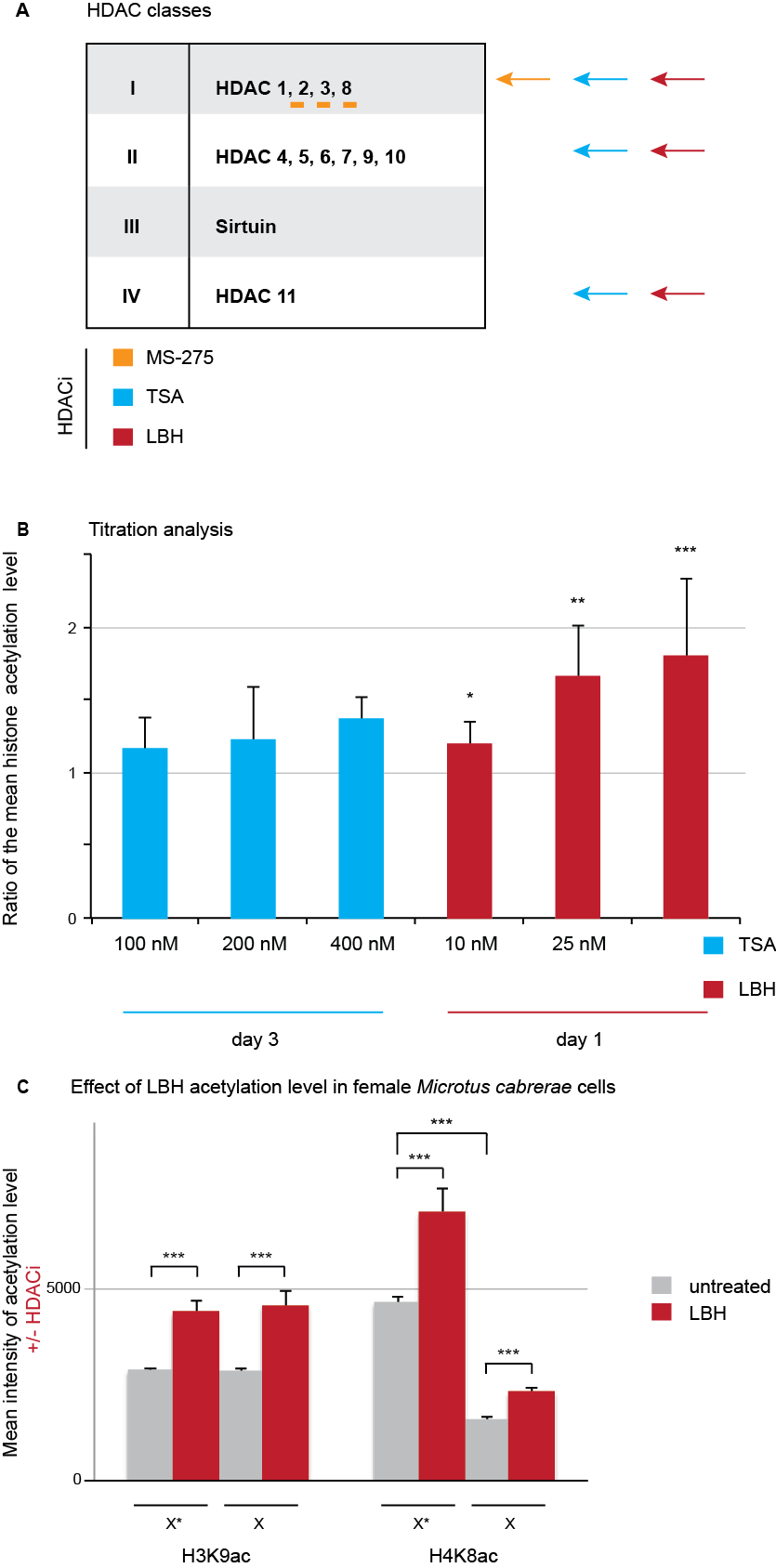

Supplement: Supplementary file 6 — Additional file 6. Titration analysis of potential HDAC inhibitors. (A) Overview of different HDAC classes and corresponding HDAC inhibitors of each class. MS-275 only affects HDAC1, HDAC2 and HDAC3 of class I (orange), whereas TSA and LBH-589 inhibit HDACs of class I, II and IV (blue/red). (B) Titration analysis of histone hyperacetylation in response to different HDAC inhibitors in female Microtus cabrerae cells. TSA treatment was performed over three days (72 hours). Previous studies have shown that a concentration of 20 nM was sufficient to hyperacetylate very condensed constitutive heterochromatin in C2C12 mouse cells [9]. Here, we increased this concentration 5x, 10x and 20x and did not achieve significant hyperacetylation at heterochromatic sex chromatin of Microtus cabrerae cells. Over time and at higher concentrations of TSA, cells showed morphological changes and with 800 nM cells died. In contrast, treatment with LBH-589 leads to hyperacetylation at sex chromatin already after one day of treatment. We were able to achieve significant increase in histone acetylation levels at the sex chromatin. To ensure a sufficient and stable hyperacetylation in the absence of morphological changes, we treated the cells with 50 nM for one day. MS-275 data are not shown as cells either showed same levels as controls or died directly after treatment. Statistical significance was tested using the t-test, comparing untreated and HDACi-treated cells. Error bars demonstrate 95Cl. ***P < 0.001. (C) Effect of HDACi treatment on histone acetylation level of female Microtus cabrerae cells. Treatment with LBH-589 leads to significantly increased H3K9ac and H4K8 mean acetylation level at sex chromatin. Mean H4K8ac level at untreated samples is significantly higher at the X* in comparison with the X. Statistical significance was tested using the t-test, comparing HDACi-treated cells and untreated cells, as well as mean H4K8ac levels in untreated cells at X* versus X. Error bars demon [file 13072_2019_262_MOESM6_ESM.png]

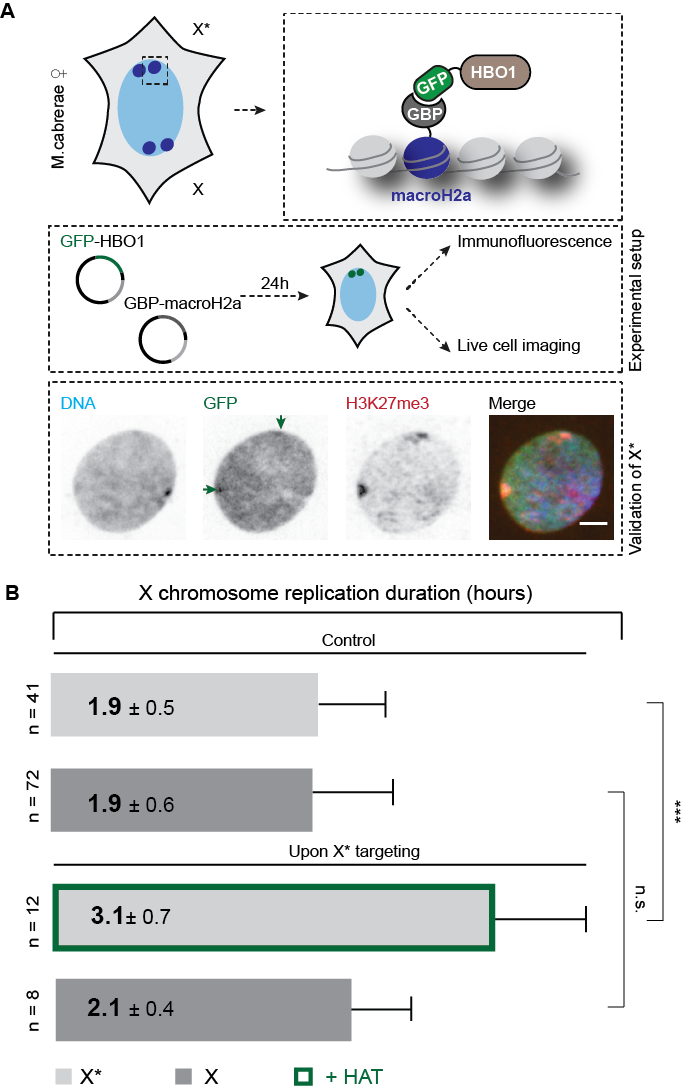

Supplement: Supplementary file 7 — Additional file 7. HBO1 targeting to the heterochromatic block of the H3K27me3 decorated X chromosome leads to prolongation of its DNA replication duration. (A) Schematic representation of targeting assay to specifically target HBO1 to the heterochromatic block of the H3K27me3 enriched X chromosome. Cells were transiently transfected with GFP-tagged HBO1 and GBP-macroH2A1. Upon co-expression, both counterparts of the targeting assay strongly interact, leading to the targeting of HBO1. Targeting was validated by immunostaining with H3K27me3 antibody as a hallmark of facultative heterochromatin. DNA (blue), GFP-HBO1 (green), H3K27me3 (red) and merge as an overlay of all three channels. Scale bar 5 µm. (B) Site-directed targeting of HBO1 to the H3K27me3-enriched X chromosome leads to a significant prolongation of DNA replication duration of the X*. Specificity of the targeting approach was validated by the fact that there was no significant difference in H3K9me3 decorated X replication duration upon X* targeting. [file 13072_2019_262_MOESM7_ESM.png]

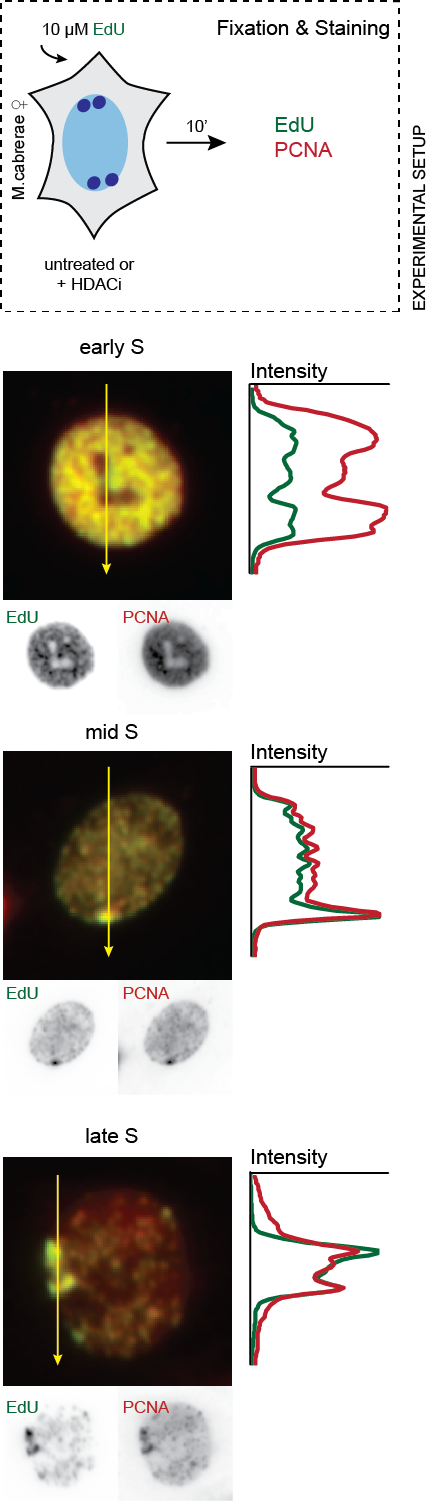

Supplement: Supplementary file 8 — Additional file 8. Demonstration of ratiometric analysis of nucleotide incorporation rate in Microtus cabrerae control cell. Modified nucleotides were added to proliferating populations of Microtus cabrerae cells and were incorporated for 10 minutes. Cells were fixed, and EdU was detected via ClickIT chemistry. PCNA was stained with an antibody. Exemplary images of an early S-, mid-S- and late S-phase cell are shown: EdU (green), PCNA (red) and merge of both channels. Line profiles depict the ratio of EdU (synthesized DNA, green) and PCNA (DNA replication machinery, red). Scale bar 5 µm. [file 13072_2019_262_MOESM8_ESM.png]

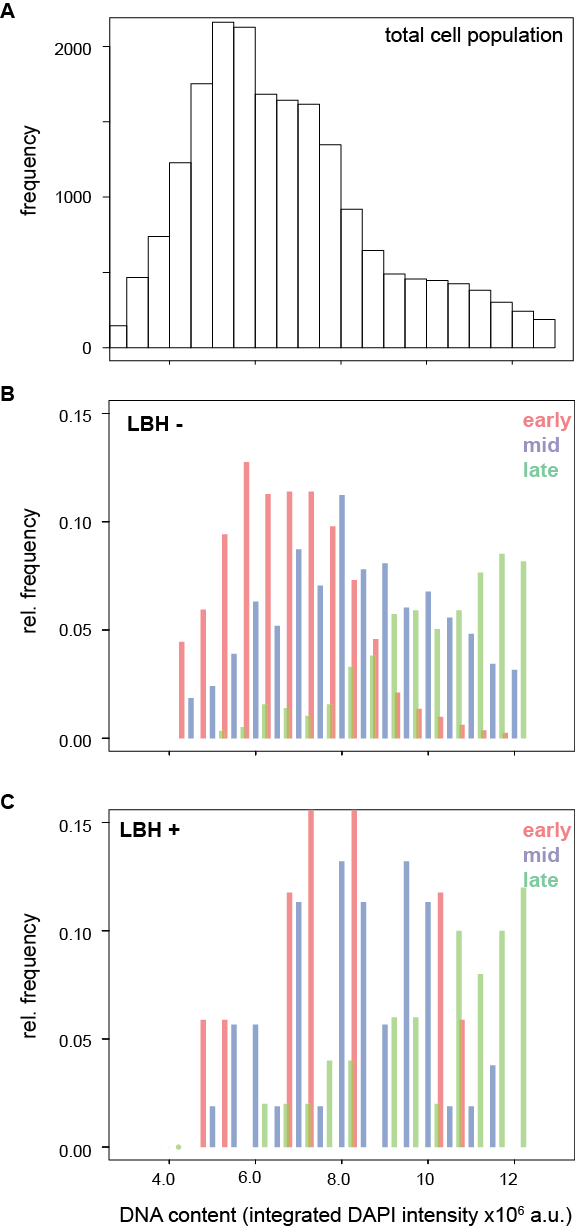

Supplement: Supplementary file 9 — Additional file 9. DNA content analysis independent of S-phase pattern staging. The data shown in Figure 7A were binned using the DNA content of the total cell population (A) to test for user-dependent S-phase pattern classification biases. Then, the frequency of the S-phase patterns assigned was plotted per DNA content bin. The early (red), mid (blue) and late (green) S-phase patterns corresponded well to the progression of genomic DNA content increase during S-phase in untreated cells (B). In contrast, in LBH-treated cells the distribution of the early replication pattern occurred within cells with a higher DNA content (red), while the late replication pattern appeared unaffected. [file 13072_2019_262_MOESM9_ESM.png]

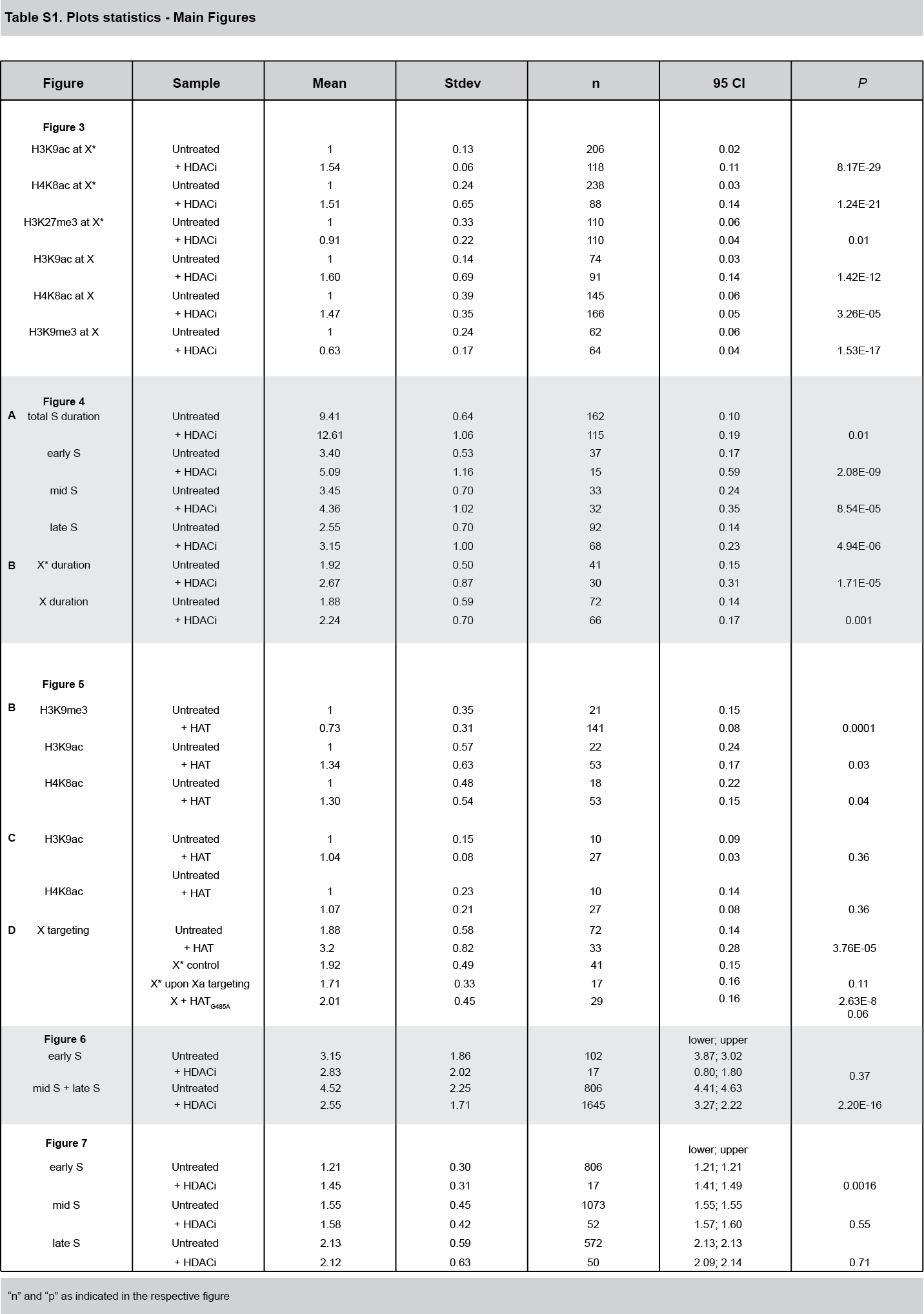

Supplement: Supplementary file 10 — Additional file 10. Plot statistics of main figures. Overview of results and statistics of data implemented in the main figures. [file 13072_2019_262_MOESM10_ESM.png]

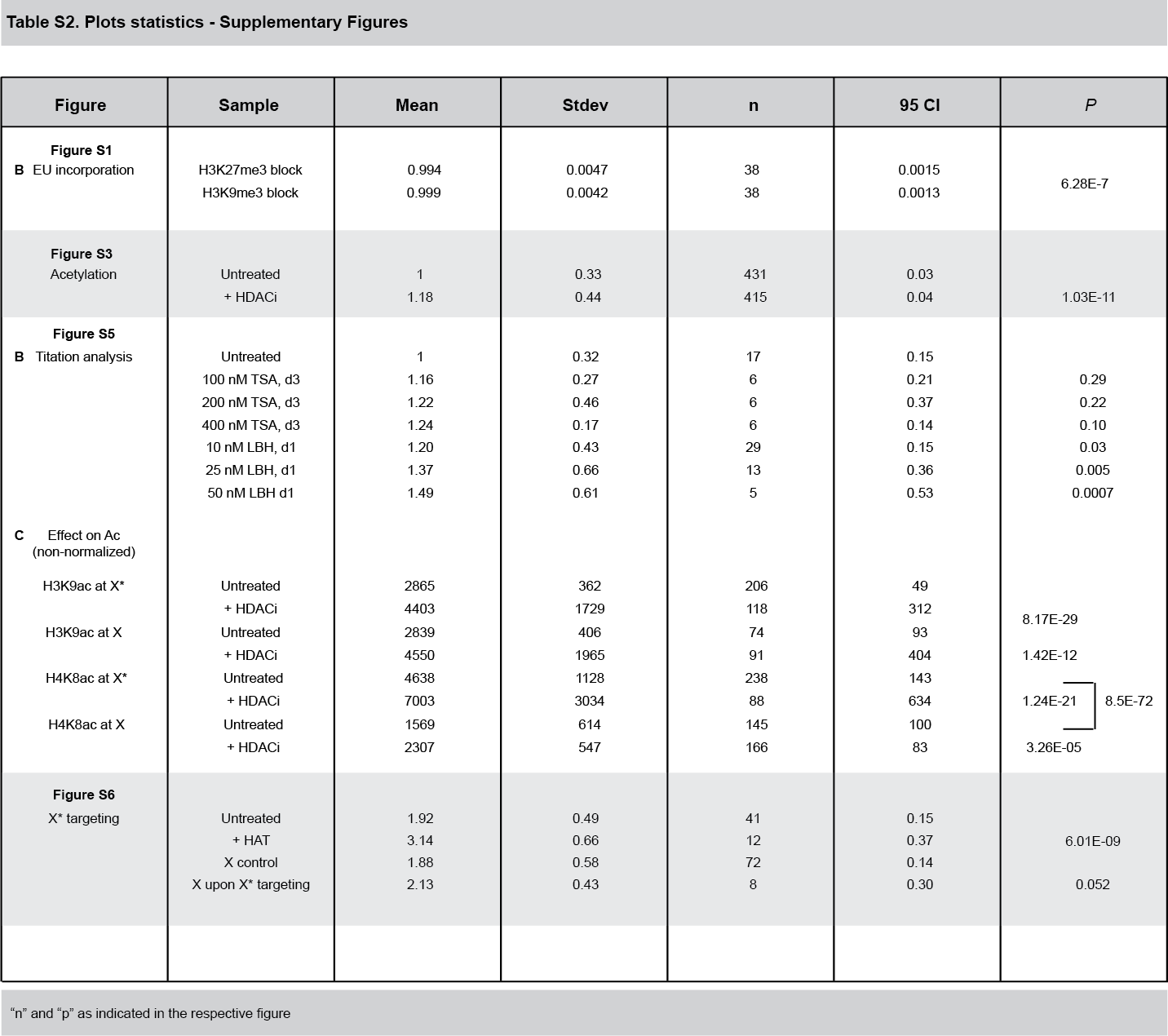

Supplement: Supplementary file 11 — Additional file 11. Plot statistics of Supplementary Figures. Overview of results and statistics of data implemented in the additional files. [file 13072_2019_262_MOESM11_ESM.png]
